# Regulation of PIEZO1 channel force sensitivity by interblade handshaking

**DOI:** 10.1126/sciadv.adt7046

**Published:** 2025-06-13

**Authors:** Katie A. Smith, Eulashini Chuntharpursat-Bon, Oleksandr V. Povstyan, Marjolaine Debant, Jacob A. Kinsella, Charlotte Revill, Charline Fagnen, Jian Shi, Richard Foster, David J. Beech, Antreas C. Kalli

**Affiliations:** ^1^ Leeds Institute of Cardiovascular and Metabolic Medicine, School of Medicine, University of Leeds, Leeds, LS2 9JT, UK.; ^2^ School of Chemistry, Faculty of Engineering and Physical Sciences, University of Leeds, Leeds, LS2 9JT, UK.; ^3^ Astbury Centre for Structural Molecular Biology, University of Leeds, Leeds, LS2 9JT, UK.

## Abstract

PIEZOs form trimeric calcium-permeable nonselective cationic channels that serve mechanical sensing needs across eukaryotic biology. Forces act on the channels by causing their curved blades to flatten and decompact, leading to an activated state, but it is unclear how this is regulated to enable the channels to adapt to different contexts. To identify potential mechanisms, we performed coarse-grained and all-atom molecular dynamics simulations on human PIEZO1. We observed an interblade handshake interaction mediated by basic amino acid residues in two flexible helices coordinated with regulated anionic lipid phosphatidylinositol 4,5-bisphosphate. The interaction determined the resting configuration of the channel, blade curvature, compactness, and ion pore structure. In experiments, disruption of the handshake by neutralization of helix amino acids or phosphatidylinositol 4,5-bisphosphate depletion increased the channel’s sensitivity to membrane tension. Structural and amino acid sequence analysis for multiple PIEZOs predicted helix amino acid arrangements for varied handshaking intensity. We suggest a dynamic interaction in PIEZO channels that regulates force sensitivity.

## Teaser

An interblade handshake interaction regulates PIEZO sensitivity to force.

## INTRODUCTION

Mechanical forces interact with wide-ranging biological phenomena, determining key biological outcomes. The identification of PIEZO channels as high-fidelity and high-sensitivity sensors of mechanical forces has improved our understanding of how such interaction works at a molecular level (*1*–*3*). PIEZO channels are evolutionarily conserved with orthologs identified in vertebrates, invertebrates, plants, and protozoa (*1*). PIEZO channels are therefore responsible for mechanosensation in diverse biology, including blood flow sensing in mammals (*4*), heart valve formation in fish (*5*), nociception in flies (*6*), root penetration in plants (*7*), and pressure sensing in pseudopods (*8*).

PIEZO channels function to permeate cations in response to mechanical stimuli (*2*). Structural studies of the mouse PIEZO1 (mPIEZO1) channel have shown that three homomeric PIEZO1 proteins assemble to form a functional molecular machine. This machine adopts a triskelion structure with extended propeller-like blades which extend out to the membrane from a central ion pore. The PIEZO1 blades are formed of repetitive transmembrane helical units (THUs) (*9*–*11*). Modeling studies suggest that this THU structure continues into the unresolved N-terminal regions, extending the blades (*12*). The highly curved structure of PIEZO1 imposes a large structural perturbation on the surrounding membrane, referred to as the “membrane footprint,” resulting in local curvature of the membrane (*9*, *12*, *13*).

Upon activation, PIEZO1 channel has been shown to undergo a conformational change to a flatter open structure (*14*–*16*), which would become increasingly energetically favorable with increased membrane tension (*9*). In silico methodologies have facilitated the study of PIEZO1 membrane mechanics under applied membrane tension (*15*, *17*). Despite the recent progress in understanding PIEZO1 activation, the mechanism by which PIEZO channels achieve context-dependent sensitivity to mechanical forces remains unknown. It is possible that the unresolved N-terminal blade regions could be important in regulating force sensitivity because recent data suggest high flexibility in these regions and their deep projection into the surrounding membrane (*18*). In this study, we began by predicting the structures of these regions in a model of the human PIEZO1 (hPIEZO1) channel equilibrated in a bilayer of endothelial lipid composition, which is one of the native contexts of PIEZO1 channels (*4*). We used this structure in conjunction with molecular simulations and lab-based methodologies to explore how an interblade interaction between adjacent PIEZO1 subunits regulates channel sensitivity to force.

## RESULTS

### Model of full-length hPIEZO1

**Fig. 1.**
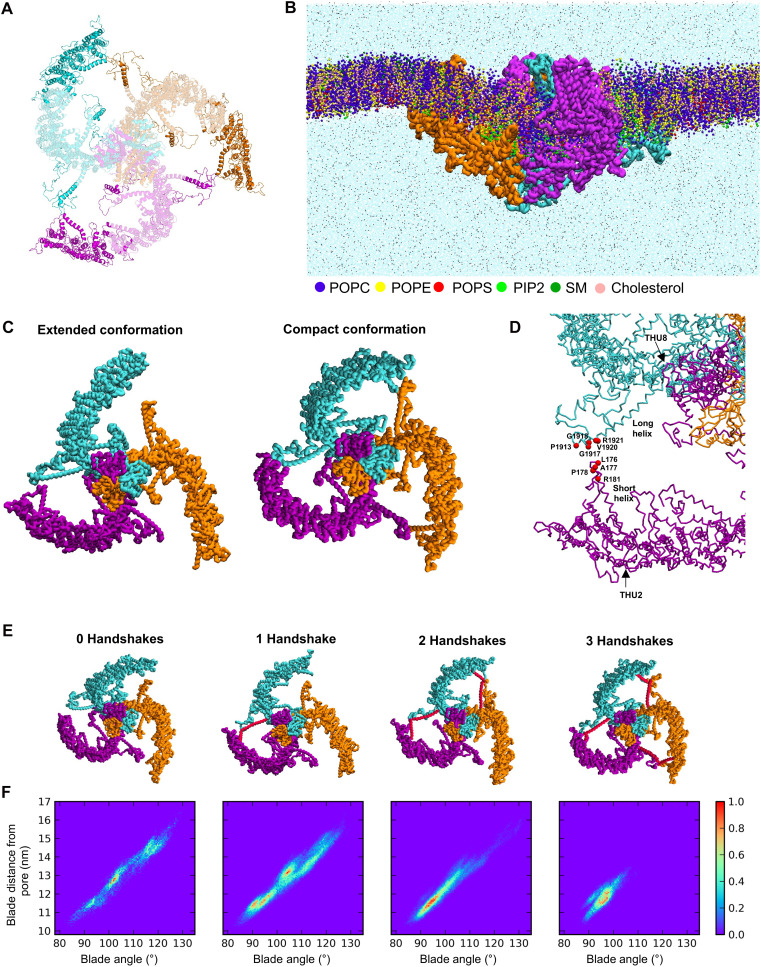
Interblade handshake in a computer model of hPIEZO1 channel. (**A**) Full-length structural model of hPIEZO1 channel shown in a cartoon representation with chains (one chain per one hPIEZO1 protein) in orange, purple, and cyan shown from above. Regions resolved in cryo-EM studies of mPIEZO1 channel are shown in light color, and unresolved regions modeled are shown in darker color (see Materials and Methods). (**B**) Snapshot taken from a trajectory for the hPIEZO1 channel simulated in an endothelial model membrane at coarse-grained resolution. hPIEZO1 backbone is shown in QuickSurf representation. Bilayer lipids are shown in Van der Waals representation: phosphatidylcholine (POPC; blue), phosphatidylethanolamine (POPE; yellow), phosphatidylserine (POPS; red), PIP_2_ (green), sphingomyelin (SM; dark green), and cholesterol (pink). Water molecules (light blue) and ions (Na^+^, Cl^−^, and Ca^2+^; black) are shown in points representation. (**C**) hPIEZO1 structures extracted from simulation trajectories adopting extended (left) and compact (right) conformations. hPIEZO1 is shown in QuickSurf representation. hPIEZO1 chains are shown in orange, magenta, and cyan. (**D**) Site of handshake interaction between two blades, highlighting two helices that are part of this interaction. hPIEZO1 chains are shown in dynamic bonds representation. Amino acid residues involved in the interaction when the handshake is formed, identified by protein contact analysis, are shown as red beads. (**E**) hPIEZO1 structures extracted from the simulation trajectory in which 0, 1, 2, and 3 handshake interactions are formed. (**F**) 2D histogram of the distance between the N-terminal THU1 and the pore (blade distance from the pore) and the angle formed by the blade. Histograms were calculated separately for frames in which hPIEZO1 forms 0, 1, 2, and 3 handshakes [aligned to images in (E)].

Our three-dimensional (3D) full-length structural model of hPIEZO1 channel is shown in Fig. 1A. The predicted N-terminal regions, unresolved by cryo–electron microscopy (cryo-EM) in mPIEZO1 (*9*–*11*) or hPIEZO1 (*19*), extend the curved blades. Comparison of this hPIEZO1 model with both the structure of mouse PIEZO2 (mPIEZO2) (*20*) and the hPIEZO1 AlphaFold2 model (AF-Q92508-F1-v4) shows good agreement and highlights that the overall structures are similar, with the N termini extending the blades (fig. S1, A to C). The N-terminal regions of the hPIEZO1 AlphaFold2 model are elevated relative to the plane defined by the pore, similar to observations in the PIEZO2 cryo-EM structure (*20*). PIEZO blades are flexible (*14*) and adopt varying conformations (*12*), so both structures may exist within the PIEZO conformational ensemble in vivo*.*

### Conformational substates associated with interblade handshaking

The hPIEZO1 channel model described above was simulated using coarse-grained molecular dynamics (CG-MD) simulations in a complex asymmetric bilayer with lipid headgroup composition selected to mimic the physiological endothelial membrane (Table 1 and Fig. 1B) (*21*, *22*). During the simulations, the hPIEZO1 channel alters the curvature of its local membrane, creating an inverted membrane dome (fig. S3), similar to the dome observed for the mPIEZO1 channel (*9*). The membrane footprint extends beyond the radius of the channel, with a convoluted trilobate topology, which creates elevated regions of the bilayer that radiate out from the blades with depressed regions in between (fig. S3A), again similar to observations for mPIEZO1 (*12*).

**Table 1.** Membrane lipid composition for endothelial membrane systems with varying phosphoinositide composition. POPC, phosphatidylcholine; POPE, phosphatidylethanolamine; POPS, phosphatidylserine; CHOL, cholesterol; PIP_2_, phosphatidylinositol 4,5-bisphosphate; DPSM, sphingomyelin.

System	Lower leaflet lipid composition (%)					Upper leaflet lipid composition (%)			
	POPC	POPE	POPS	CHOL	PIP_2_	POPC	POPE	DPSM	CHOL
**WT hPIEZO1**									
Endothelial membrane	50	20	5	20	5	55	20	5	20
0% PIP_2_	50	20	10	20	0	55	20	5	20
**Mutant (SHM) hPIEZO1**									
Endothelial membrane	50	20	5	20	5	55	20	5	20

The simulated blades show extensive motions in the bilayer plane that are asymmetric, with the three blades moving apparently independently of one another (Fig. 1C). The channel fluctuates between extended and compact conformations (Fig. 1C). Compact conformation coincides with the interaction between the membrane parallel helix associated with THU2 [referred to as the short helix (SH)] of one blade and the membrane parallel helix associated with THU8 [referred to as the long helix (LH)] of the neighboring blade of the same channel (Fig. 1D and movie S1). Protein contact analysis reveals that this interaction occurs primarily between the peripheral ends of the SH and LH (Fig. 1D and fig. S4). We refer to this interaction as the interblade handshake.

In the compact conformation, all three blades form handshakes, while the extended conformations form 0, 1, or 2 handshakes (Fig. 1E). To gain insight into the effect of the number of handshake interactions formed on blade dynamics, we extracted simulation frames associated with 0, 1, 2, and 3 handshakes and measured the distance between the first N-terminal THU (THU1) and the pore and the angle formed by the blades (see Materials and Methods) (Fig. 1F). Channels adopting 0, 1, and 2 handshakes exhibit wide variations in distance and angle from 10.5 to 16.5 nm and 85° to 130°. As the number of handshakes increases, there is more sampling of conformations associated with compact blade structures, i.e., distance < 13 nm and angle < 100° (Fig. 1F). Conformations associated with three handshakes show relatively little blade movement, with all blades forming compact structures. The handshakes, therefore, afford the hPIEZO1 blades conformational flexibility by varying the number of interactions, thereby altering how dynamic the structure is.

### Handshaking involves phosphatidylinositol 4,5-bisphosphate

**Fig. 2.**
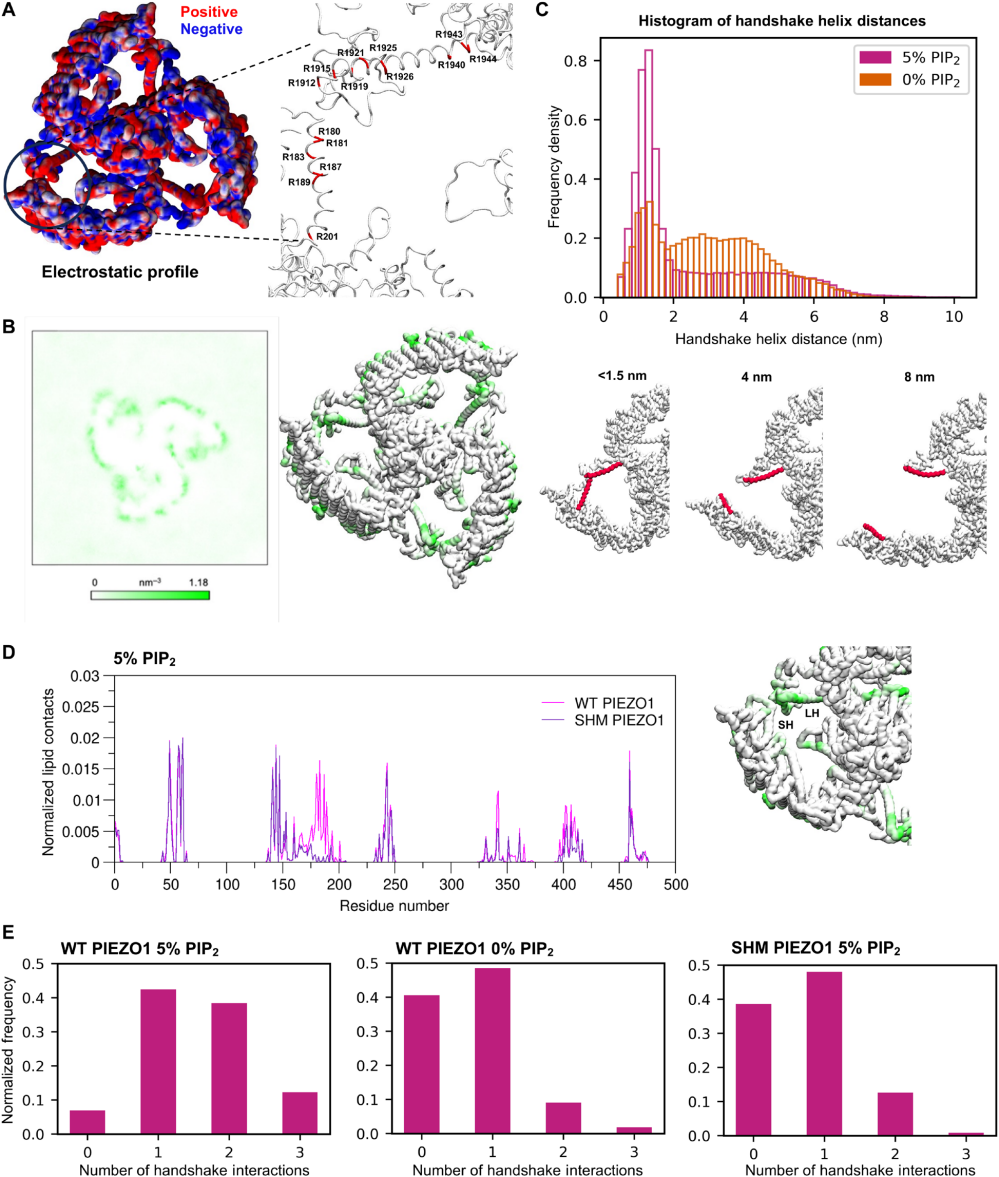
Involvement of PIP_2_ in the handshake. (**A**) Electrostatic profile of hPIEZO1 mapped onto hPIEZO1 structure (left). Arginine residues on the handshake helices (right). (**B**) PIP_2_ density averaged across five repeat simulations (left). PIP_2_ contacts mapped onto PIEZO1 structure (right). PIEZO1 backbone is colored according to the number of PIP_2_ contacts with no contacts shown in white and high number of contacts in green. (**C**) Histogram of distances between the final residue of each helix which forms the handshake interaction for hPIEZO1 simulated in an endothelial membrane containing 5% PIP_2_ (pink) and 0% PIP_2_ (orange) (top). Example structures were extracted corresponding to helix tip separation distances of 1.5, 4, and 8 nm with helices in red (bottom). (**D**) PIP_2_ contacts with the SH of the handshake for wild-type (WT) hPIEZO1 (pink) and the SH mutant (SHM; purple) simulated in an endothelial membrane containing 5% PIP_2_. PIP_2_ contacts were mapped to the SHM structure with no contacts shown in white and high number of contacts shown in green. PIP_2_ contacts are abolished on the SH, where the mutations were made, compared to the LH and the WT channel shown in (B) (right). (**E**) Frequency of the 0, 1, 2, and 3 handshake conformations for WT hPIEZO1 simulated in the presence of PIP_2_ (left) or absence of PIP_2_ (center) and SHM hPIEZO1 simulated in the presence of PIP_2_ (right).

Assessment of the electrostatic profile of hPIEZO1 (Fig. 2A) highlights that both membrane parallel helices of the handshake interaction contain a high density of positively charged (arginine) residues, favoring repulsion rather than the interaction. We, therefore, hypothesized the involvement of an anionic lipid interface. Analysis of hPIEZO1/lipid interactions in the model predicts the anionic phosphatidylinositol 4,5-bisphosphate (PIP_2_) lipid as the predominant interacting species (fig. S5A). PIP_2_ density forms an anionic annulus around the PIEZO1 blades, and when the PIP_2_ contacts are mapped to the hPIEZO1 structure, contacts with PIP_2_ are evident at the handshake region (Fig. 2B). Therefore, our simulations suggest that PIP_2_ is required to neutralize the positive charges, enabling the neighboring blades to come together and form a compact hPIEZO1 structure.

To assess the role of PIP_2_ in the handshake, hPIEZO1 was simulated in a model membrane without PIP_2_. The effect on the frequency of handshake formation was calculated by measuring the distance between the final amino acid residue of each of the handshake helices over simulation time. In the presence of PIP_2_, hPIEZO1 spends most of the simulation time in conformations in which at least one handshake interaction is formed with relatively little time spent in conformations associated with a handshake helix distance of greater than 2 nm (Fig. 2C). A distance of <1.5 nm is associated with handshaking. Consistent with the proposed role of PIP_2_, in its absence, the proportion of simulation time in which at least one handshake forms is reduced. hPIEZO1 structures associated with different handshake helix distances were extracted from the simulation trajectory (Fig. 2C). Without PIP_2_, handshakes still form but with reduced frequency (Fig. 2C). Phosphatidylserine, another anionic lipid, now interacts with the channel at the handshake sites (figs. S5B and S6D). In ~7% of simulation frames in the system with 0% PIP_2_, one chain adopts conformations in which both the blade distance from the pore and the blade angle are less than those observed for the handshake states (fig. S6A). While this appears to indicate formation of a more compact state than that afforded by the three-handshake state, this is not the case. Visualization of these frames reveals that one chain adopts a conformation in which the blade is more curved compared to the other two blades, thereby bringing the end of the blade closer to the pore, independent of handshaking (fig. S6, B and C).

### SHM reduces handshake interaction and increases blade dynamics

To further investigate the idea of handshaking, a mutant hPIEZO1 model [referred to as the SH mutant (SHM)] was generated in which the putative PIP_2_ interacting amino acids (R180, R181, R183, R187, and R189) on the SH were mutated in silico to alanine. This SHM hPIEZO1 channel was simulated in the same complex bilayer. Analysis of hPIEZO1 lipid contacts confirms that the mutations resulted in the disruption of PIP_2_ interaction at the mutation sites with little or no effect on PIP_2_ and other lipid interactions at other sites on the hPIEZO1 structure (Fig. 2D and fig. S5, C and D).

This localized disruption of PIP_2_ interactions was sufficient to destabilize the handshake interaction, resulting in a reduced population of conformations associated with a helix-helix distance of <1.5 nm following simulations for 3 or 5 μs (fig. S7A). Comparison of the frequency of 0, 1, 2, and 3 handshake conformations for each system confirms that the localized disruption of PIP_2_ interactions was sufficient to decrease the frequency of 2 and 3 handshake conformations compared to the system with the wild-type (WT) hPIEZO1 (Fig. 2E). As a result, the SHM channel spends more time in conformations associated with extended blade structures (fig. S6B compared with Fig. 1F). A similar decrease in 2 and 3 handshake conformations was observed in the system with 0% PIP_2_ (Fig. 2E). The SHM channel also adopts conformations in which both the blade distance from the pore and the blade angle are less than those observed for the handshake states similar to those described above (fig. S7B compared to fig. S6A). Therefore, the arginine-to-alanine mutations in the SH create a channel that, in the presence of PIP_2_, is similar to the WT channel in the absence of PIP_2_.

### Handshake stability correlates with PIP_2_ density

Comparison of PIP_2_ interactions between the 0 and 3 handshake states in our simulations allows us to understand how the movement of the handshake helices alters PIP_2_ interactions. PIP_2_ interacts with the handshake helices in all conformations including when the handshake is released (fig. S8). When a handshake is formed, the PIP_2_ interactions localize to the site of the handshake interaction, i.e., residues which form the middle-to-peripheral end of the helix (residues 180 to 190), resulting in a large PIP_2_ lipid pool around the interaction site (fig. S8). When the handshake is released, the PIP_2_ interactions are more dispersed along the helix. The change in angle of the SH when the handshake is formed compared to that when the handshake is released sterically blocks interactions from occurring closer to the blade domain, resulting in loss of interaction with residue 201 (fig. S8). This also occurs to a lesser extent for the LH, which is less dynamic and so does not undergo a large change in orientation.

**Fig. 3.**
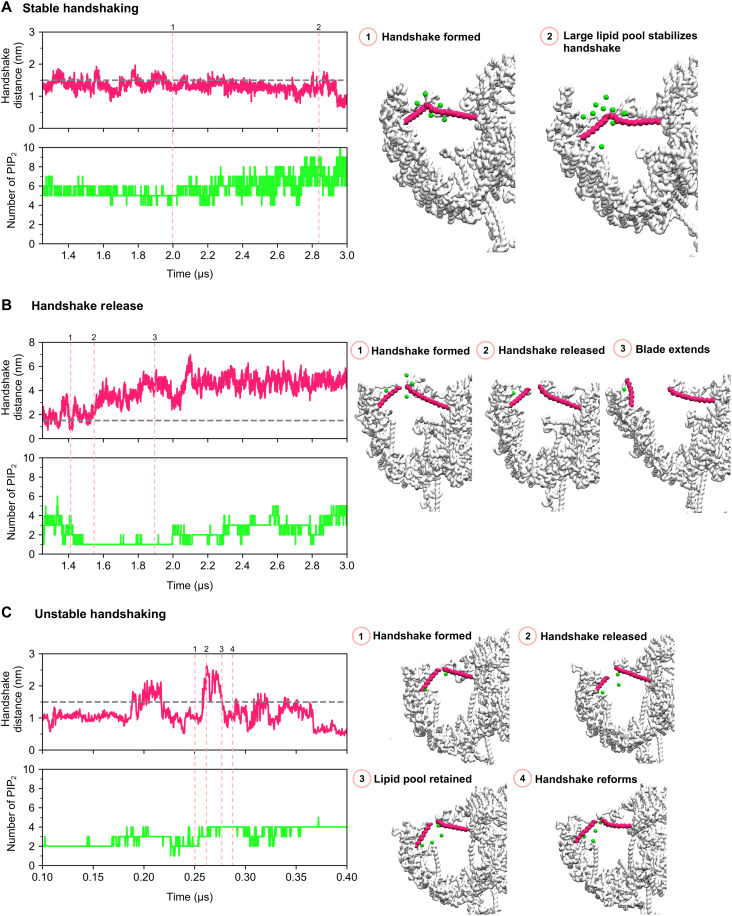
Handshaking occurs in modes that depend on PIP_2_ distribution. The handshake distance (between the final residues of each helix involved in the handshake interaction) and the number of PIP_2_ lipids within 2.5 nm of the SH of the interaction were plotted over simulation time. On the left, example plots characteristic of stable handshaking (**A**), handshake release (**B**), and unstable handshaking (**C**). On the right, snapshots associated with the distinct stages of each handshake mode. The backbone of hPIEZO1 is shown in surface representation in white. The handshake helices are pink. Each green bead corresponds to the headgroup of one PIP_2_ lipid. These plots are examples that were identified from the plots of the complete simulations shown in figs. S9 to S13.

To visualize handshake dynamics, we calculated the number of PIP_2_ molecules within 2.5 nm of the SH. When visualized with handshake distance over time, dynamics of the handshake and PIP_2_ are seen (figs. S9 to S13). Examples of the dynamic motions associated with changes in PIP_2_ density, identified in the replicate simulations, are shown in Fig. 3. A large pool of PIP_2_ stabilizes the handshaking (Fig. 3A). Handshake release occurs when there are fewer PIP_2_ molecules (Fig. 3B). If there are still sufficient PIP_2_ molecules around after release, the handshake reforms but dissociates repeatedly on short timescales, resulting in unstable handshaking (Fig. 3C). The data suggest a PIP_2_ pool that determines the handshake stability. These data suggest that PIP_2_ at the handshake interaction site is fundamental for stabilizing the handshake interaction.

The SHM diminishes the PIP_2_ sites at the SH; however, our models show that handshaking still occurs (Fig. 2E). The loss of the PIP_2_ interaction sites on the SH would render stable handshaking impossible, indicating that when the SHM hPIEZO1 forms handshake interactions, it is likely to be in the unstable mode. Together, this suggests that SHM hPIEZO1 exhibits increased mechanical sensitivity due to both the decrease in and the destabilization of interblade handshaking.

### SHM decreases the depth of the dome

Analysis of the depth of the dome revealed that the SHM channel imposes a shallower membrane dome (fig. S3, A and B), which is consistent with its being less compact. Calculation of the area that the channel covers within the membrane (projected area) associated with 0, 1, 2, and 3 handshake states reveals that the projected area increases with decreased handshake interactions, indicating that handshake release also results in channel in-plane area expansion in the absence of pressure (fig. S3C). Both the depth of the dome and in-plane area expansion of the membrane-channel system have been suggested to be linked to PIEZO mechanosensitivity (*9*). Therefore, our data are consistent with the idea that handshaking is likely to be a mechanism for regulating the mechanical sensitivity.

### SHM enhances openness of the pore region

To understand how disruption of the handshaking alters the structural rearrangements in response to applied membrane tension, we converted our CG models to atomistic (AT) resolution and simulated the WT and SHM channels in the presence of −30- and 1-bar pressure applied equally in the *x* and *y* dimensions to simulate membrane tension and no tension, respectively.

**Fig. 4.**
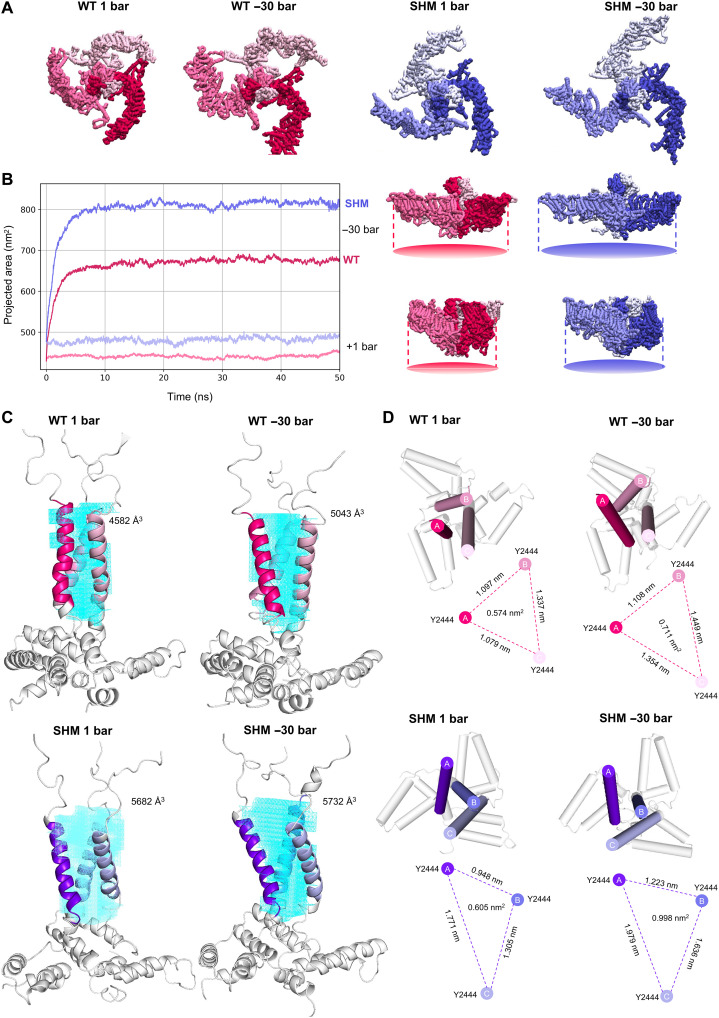
Effect of interblade handshaking on PIEZO1 conformational changes in response to applied tension. A snapshot from CG-MD simulations of WT and SHM hPIEZO1 simulated in a model endothelial membrane containing 5% PIP_2_ was converted to AT resolution and simulated in the presence of (−30 bar) and the absence of (+1 bar) tension. (**A**) Final snapshots following AT simulations under each condition are shown for WT (pink) and SHM (purple) hPIEZO1 from the top view. Backbones of the channels are shown in surface representation. Each chain is shown in a different shade of pink for WT and purple for SHM. (**B**) Projected area of the channel was calculated over simulation time WT (pink) and SHM (purple) hPIEZO1 (left). Data for 1- and −30-bar simulations are shown in light (1 bar) and dark (−30 bar) colors. Final snapshots of WT and SHM hPIEZO1 are shown from side view and include depictions of the projected area under each condition at 50 ns (right). (**C** and **D**) Characterization of the inner cavity of the pore for WT (pink) and SHM (purple) hPIEZO1 following AT simulation for 50 ns at 1 and −30 bar. Residues 2424 to 2521 are shown in cartoon representation. The inner pore helix residues (residues 2438 to 2458) are colored in shades of pink for WT and purple for SHM. Regions which do not form the inner helix are white and transparent. (C) The pore is shown from the side view. Volume of the pore was calculated using trjcavity and is shown in cyan. (D) The pore is shown from the top view. The area of the triangle formed by residue Y2444 in each chain is depicted below each snapshot.

The AT simulations revealed that, in the basal state at 1 bar, SHM hPIEZO1, while still curved, adopts a more extended structure with an increased projected area compared to the WT channel (Fig. 4, A and B). In response to −30-bar pressure, both WT and SHM hPIEZO1 flatten and expand, resulting in decrease in blade height (fig. S14) and an increased projected area (Fig. 4B). Both channels achieve the same flatness, but SHM hPIEZO1 undergoes greater expansion in the *xy* plane compared to the WT channel (Fig. 4, A and B). Blade expansion correlates with channel activation (*18*), leading to the prediction that SHM should increase channel activation.

At the CG resolution, large-scale conformational changes cannot occur because of the elastic network. It was, therefore, not possible to observe changes in the pore between the WT and SHM hPIEZO1 channels. However, a comparison of the pore components in AT simulations at 1 bar (i.e., without tension) shows that the SHM pore is more dilated compared to the WT channel (Fig. 4C). At −30 bar, both the WT and SHM channel pores further dilate. However, the WT channel under tension does not reach a pore volume as dilated as the SHM PIEZO1 channel under no tension (Fig. 4C).

Calculation of the triangle area formed by selected residues in each chain at the top (Y2444; Fig. 4D and fig. S15), middle (V2450; fig. S16), and bottom (F2454; fig. S17) of the inner pore-lining helix TM38 shows that the SHM channel is more dilated than the WT channel at all levels (figs. S15 to S17). V2450 marks the beginning of the hydrophobic region of the pore inner helix and has been identified as a constriction point in the cryo-EM structures of mPIEZO1 (*23*). As a result, we calculated the area of the triangle defined by this region in our models. In the SHM channel, V2450 of chain C is rotated by ~45°C such that L2449 lines the inner pore (fig. S16B). To calculate the distances between the three chains and the area of the triangle defined by them at the narrowest region of the pore, we, therefore, used V2450-V2450-L2449 as the residues for this calculation in the SHM channel. Consistent with experimentally derived structures, this is the narrowest region of the pore in our WT and SHM models (fig. S16). Under tension, the SHM PIEZO1 pore becomes further dilated at the top, middle, and bottom of the pore. However, we only observe increases in the pore dimensions at the top level of the pore for the WT channel (Fig. 4D and figs. S15 to S17). Together, these data suggest that the SHM mutations that destabilize the handshake interaction also enhance the conformational rearrangements that occur in response to tension. The SHM hPIEZO1 channel undergoes a larger in-plane area expansion in response to tension and primes the pore, resulting in a more open central cavity compared to the WT channel. The data suggest a hypothesis in which interblade handshaking functions to suppress PIEZO1 mechanical sensitivity and channel activation.

The handshake concept suggests that PIEZO1 blades function independently to adopt varying conformations. Final snapshots from each simulation highlight this varied blade arrangement, which causes asymmetry in the channel structure (Fig. 4A). Analysis of the blade height of the WT and SHM hPIEZO1 blades under no tension shows that this fluctuates, further highlighting the asymmetry in these structures (fig. S14, B and C). Tension in our systems is applied uniformly in the *x* and *y* dimensions. Under tension, the three blades flatten simultaneously to a final height of ~1 nm with little variation between them (fig. S14, B and C). However, the initial asymmetry in the channel structures is conserved (Fig. 4A). In addition, the distances between the three inner helices of the pore are nonuniform, resulting in asymmetry of the pore structures. This asymmetry is more pronounced in the SHM channel, resulting in one side of the inner helix being more affected than the others (Fig. 4D and figs. S15 to S17). This suggests that the handshake state adopted by the channel determines the sensitivity to mechanical force and the conformation of the activated structure. This poses the idea that the handshake concept may regulate the degree of pore opening and exposure of specific lipid and/or protein interaction sites and subsequently play a role in regulating differential signal transduction.

### SHM enhances mechanical sensitivity

**Fig. 5.**
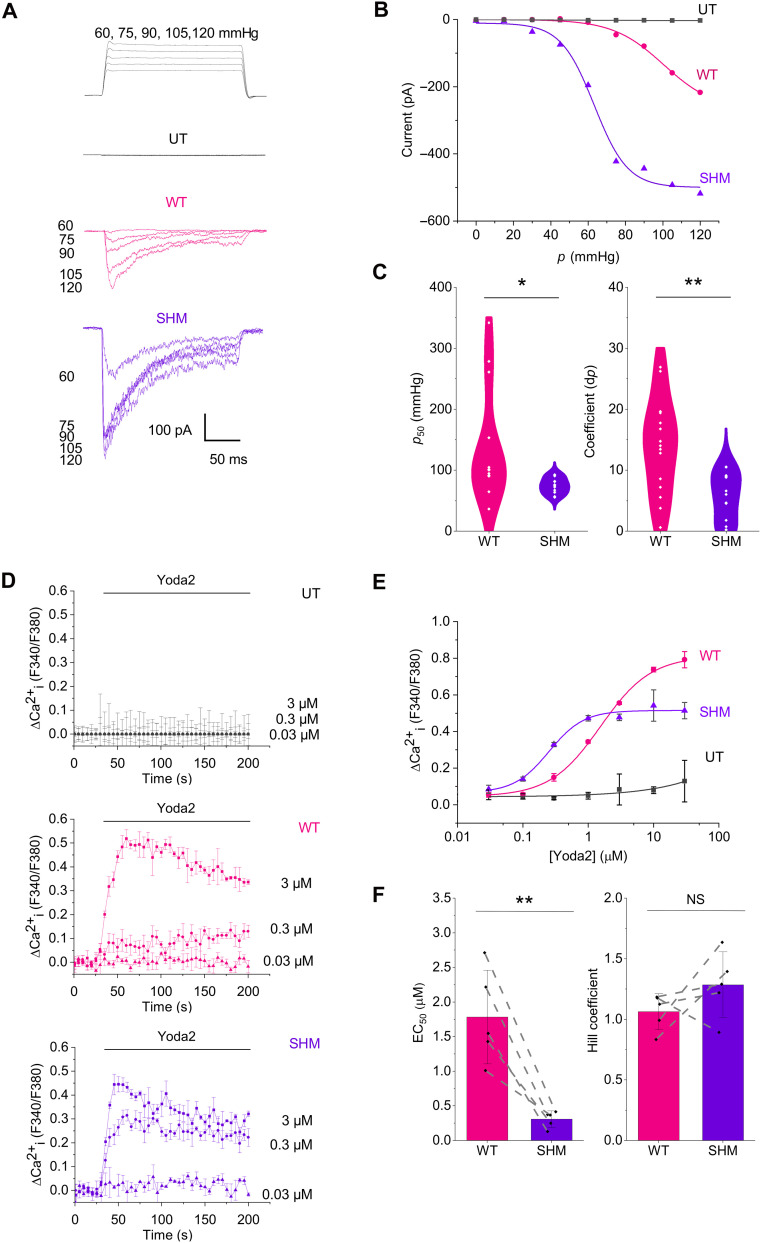
SHM enhances mechanical and agonist sensitivities. Outside-out patch electrophysiology (**A** to **C**) and intracellular Ca^2+^ measurements (**D** to **F**) in HEK293 cells expressing WT hPIEZO1, SHM hPIEZO1, or neither (UT). (A) Example current traces with pressure pulses are shown at the top. (B) Peak inward current amplitudes for the experiments of (A) plotted against pressure (*p*) and fitted using the Boltzmann function. (C) Boltzmann function parameters for all experiments of the type illustrated in (B), showing the *p* for 50% activation (*p*_50_) (left) and the Boltzmann coefficient (right). Data are represented as violin plots with individual data points superimposed (WT *n* = 15 and SHM *n* = 13 independent patch recordings). Statistical test (unpaired *t* test not assuming equal variance) probability (*P*) results were *P < 0.05 (*P* = 0.02006) and **P < 0.01 (*P* = 0.00151). Values for WT are estimates because the currents did not saturate. (D) Example UT, WT, and SHM data showing change (Δ) in intracellular Ca^2+^ in response to Yoda2 indicated by the Fura-2 fluorescence (F) ratio F340/F380. Data are means ± SEM for three wells (technical replicates) in a 96-well plate (one independent experiment each). (E) Peak ΔCa^2+^ amplitudes for the experiments of (D) plotted against Yoda2 concentration and fitted using the Hill function. (F) Hill function parameters for all experiments of the type illustrated in (E), showing the concentration of Yoda2 for 50% effect (EC_50_) (left) and the Hill coefficient (right). Data are means ± SD with individual data points superimposed (WT *n* = 5 and SHM *n* = 5 independent experiments). Dashed lines join the data points for each paired comparison of WT and SHM. Statistical test (paired *t* test) probability (*P*) results were **P < 0.01 (*P* = 0.0074) and NS (not significant, *P* = 0.15494).

To test the handshaking concept in laboratory experiments, patch-clamp electrophysiology was applied to untransfected (UT) human embryonic kidney (HEK) 293 cells and the same cells stably overexpressing WT or SHM hPIEZO1 using a blinded randomized experiment design. The SHM did not alter the amount of hPIEZO1 expressed (fig. S18). Channel currents were recorded from outside-out patches to which positive pressure pulses were applied. Small amounts of endogenous hPIEZO1 occur in UT HEK293 cells (fig. S18) (*24*), but pressure-activated currents were absent or not distinguishable from background leak currents (Fig. 5A and fig. S19A). By contrast, cells overexpressing WT hPIEZO1 produced the expected large fast-activating and inactivating inward currents that increased with incrementing pressure (Fig. 5A). The currents did not reach a maximum at a pressure of 120 mmHg, and we could not apply pressure greater than 120 mmHg because the patch was often lost, we assume because of the membrane disruption. Cells stably overexpressing SHM hPIEZO1 produced inward currents that, by contrast, increased more readily and saturated at 105 mmHg (Fig. 5A). Analysis of all recordings (see Materials and Methods) revealed that SHM hPIEZO1 is more pressure sensitive (Fig. 5, B and C, and fig. S19, A to D). The data support the idea that handshaking suppresses mechanical sensitivity of the channels.

### SHM enhances agonist sensitivity

Yoda1 is a small-molecule agonist of the PIEZO1 channel that increases channel mechanical sensitivity through a putative wedge-like interaction with the proximal blade region (*17*). We, therefore, hypothesized that handshaking might also affect agonist sensitivity. To test this hypothesis, we used Yoda2, an analog of Yoda1 with improved aqueous solubility, potency, and efficacy compared with Yoda1, thereby facilitating the construction of full concentration-response curves for hPIEZO1 channels (*25*). We again compared UT and overexpressing HEK293 cells in blinded experiments using an orthogonal assay based on measuring intracellular Ca^2+^. WT hPIEZO1 cells had the largest responses overall, but the threshold for activation was at ~0.3 μM Yoda2 and the responses did not saturate (Fig. 5, D and E). SHM hPIEZO1 cells had a lower threshold at ~0.03 μM, and the responses saturated, revealing a classical sigmoid dose-response curve (Fig.5, D and E). UT cells were largely unresponsive (Fig. 5, D and E). Analysis of all recordings (see Materials and Methods) revealed that SHM hPIEZO1 is more Yoda2 sensitive (Fig. 5F and fig. S20). Values for WT hPIEZO1 are estimates because the Ca^2+^ signals did not saturate. The data suggest that disruption of the handshake enhances agonist sensitivity, consistent with enhanced mechanical sensitivity.

### PIP_2_ depletion enhances mechanical sensitivity

**Fig. 6.**
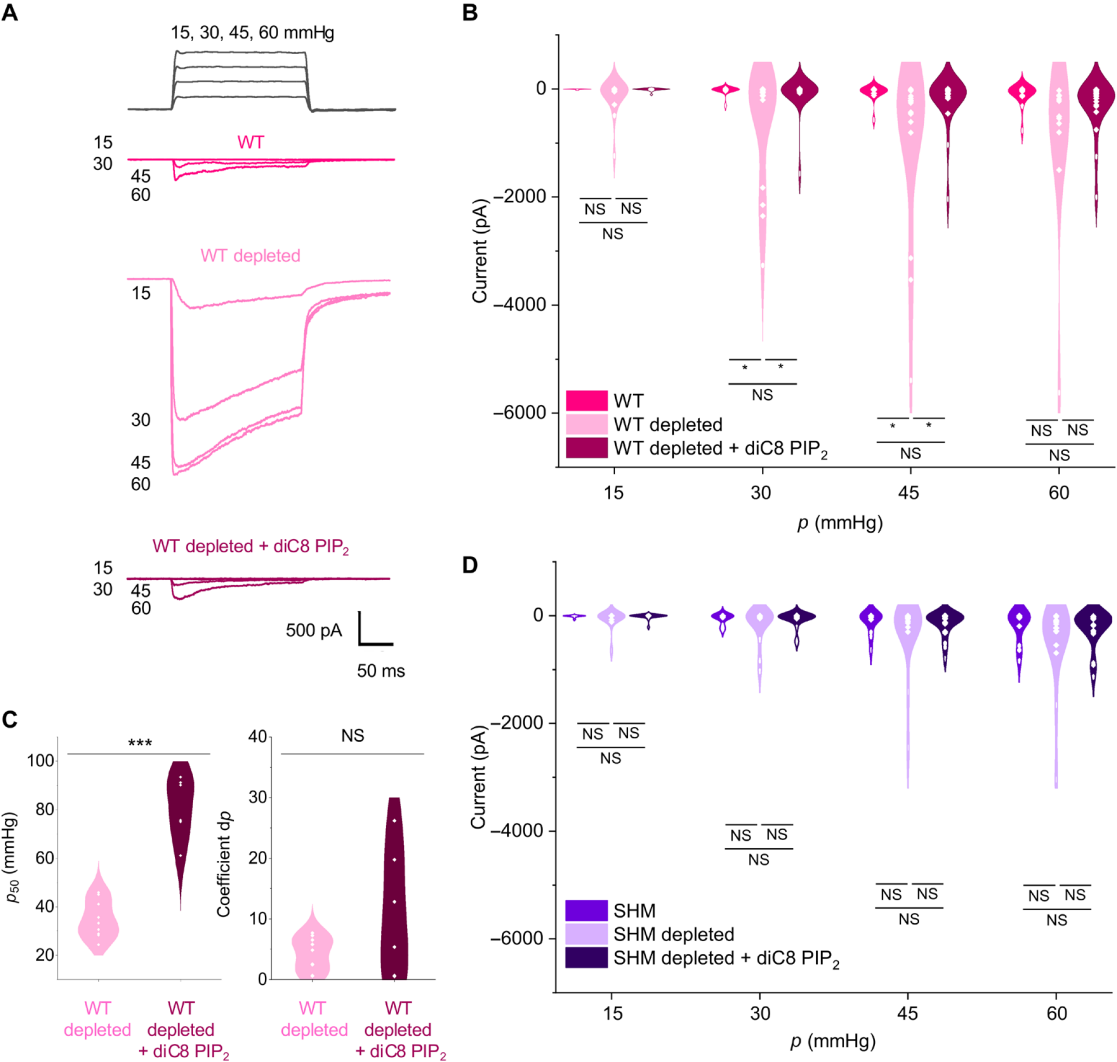
PIP_2_ depletion enhances mechanical sensitivity of WT but not SHM hPIEZO1. Outside-out patch data for HEK293 cells expressing WT or SHM hPIEZO1. (**A**) Example current traces with pressure pulses shown at the top. (**B**) Peak inward currents for experiments of the type shown in (A). WT (*n* = 15), WT PIP_2_ depleted (*n* = 15 except *n* = 11 for 60 mmHg), and WT PIP_2_ depleted + diC8 PIP_2_ (*n* = 20 except *n* = 19 for 60 mmHg) data. The statistical test was one-way analysis of variance (ANOVA) with Tukey analysis and the probability (*P*) results were NS (not significantly different, *P* > 0.05) or *(*P* < 0.05): 30 mmHg WT versus WT depleted *P* = 0.02; WT depleted versus WT depleted + diC8 *P* = 0.03; 45 mmHg WT versus WT depleted *P* = 0.02; WT depleted versus WT depleted + diC8 *P* = 0.04; 60 mmHg WT versus WT depleted *P* = 0.049; WT depleted versus WT depleted + diC8 *P* = 0.15. (**C**) Boltzmann function parameters showing the pressure (*p*) for 50% activation (*p*_50_) (left) and Boltzmann coefficient (right) for the experiments of (B). Data are means ± SD with individual data points superimposed (WT depleted *n* = 9 and WT PIP_2_ depleted + diC8 *n* = 6 independent patch recordings). Statistical test (unpaired *t* test not assuming equal variance) probability (*P*) results were ***(*P* = 6.17 × 10^−5^) and NS (not significantly different). (**D**) Similar to (B) but for SHM (*n* = 13), SHM PIP_2_ depleted (*n* = 16), and SHM PIP_2_ depleted + diC8 PIP_2_ (*n* = 22) data. The statistical test was two-sample *t* test, unpaired, unequal variance, and the probability results all indicated NS (not significantly different).

To investigate the relevance of PIP_2_, we first depleted PIP_2_ by treating cells with wortmannin to inhibit PI4K that catalyzes the production of PIP_2_ in combination with pulses of adenosine triphosphate (ATP) to activate receptors that stimulate PIP_2_ hydrolysis (fig. S21). Channel currents were again recorded from outside-out patches to which positive pressure pulses were applied (Fig. 6A). PIP_2_-depleted cells overexpressing WT hPIEZO1 showed a large increase in pressure sensitivity compared to WT hPIEZO1 in control cells (Fig. 6, A to C compared with Fig. 5, A to C). PIP_2_ depletion, by contrast, had no effect on SHM hPIEZO1 (Fig. 6D). We next sought to reintroduce PIP_2_ and thereby rescue the channel properties of WT hPIEZO1 in control cells. We used a short carbon chain form of PIP_2_ (diC8 PIP_2_) that is more water soluble than native PIP_2_ and can therefore be included in the patch pipette solution for delivery to the inner membrane leaflet. diC8 PIP_2_ returned the properties of WT hPIEZO1 largely to those seen before PIP_2_ depletion (Fig. 6, A to C, and fig. S22), but the diC8 PIP_2_ had no effect on SHM hPIEZO1 (Fig. 6D). The data support our hypothesis that handshaking involves PIP_2_.

### Conservation of handshake features in other PIEZOs

The handshake mechanism is likely to be relevant beyond hPIEZO1 channels. Analysis of CG-MD simulations on the mPIEZO1 channel, previously published by Chong *et al.* (*12*), reveals that there may be similar but modified handshaking in this PIEZO1 (fig. S23). In place of some of the arginine residues, mPIEZO1 has lysines (which are also positively charged) and fewer arginines in these regions (one lysine and five arginines in one handshake helix and five arginines and three lysines in the other). In hPIEZO1, there are six arginines in one helix and nine arginines in the other and no lysines (fig. S24C). Principles of the handshake mechanism are also evident in the mPIEZO2 channel, as shown by our model of mPIEZO2 that is based on cryo-EM data (fig. S24, A and B) (*20*). Although the handshake regions were not resolved by cryo-EM (possibly because of their high flexibility), secondary structure prediction and homology modeling suggest the existence of the handshake helices also in this PIEZO (fig. S24B). The mPIEZO2 helices contain a high density of positively charged residues, which are again different in detail compared with hPIEZO1 (fig. S24C). Sequence alignment of PIEZO orthologs across diverse eukaryotes reveals conservation of positively charged amino acid residues in putatively equivalent positions (fig. S24D). There is variability in the number and the proportion of arginines and lysines between species. Arginine is associated with a stronger preference for positive charge within lipid membranes (*26*), indicating that channels with a higher ratio of lysine to arginine at the key sites may have reduced frequency of handshaking and thereby greater mechanical sensitivity, contributing to PIEZO subtype- and species-dependent mechanovariation. Therefore, we suggest that the concept of regulation of PIEZO1 sensitivity by a handshake interaction may be broadly applicable to PIEZOs and important for the optimization of PIEZO mechanical sensitivity to mechanical needs of specific cell types, organs, organisms, and environmental contexts.

## DISCUSSION

**Fig. 7.**
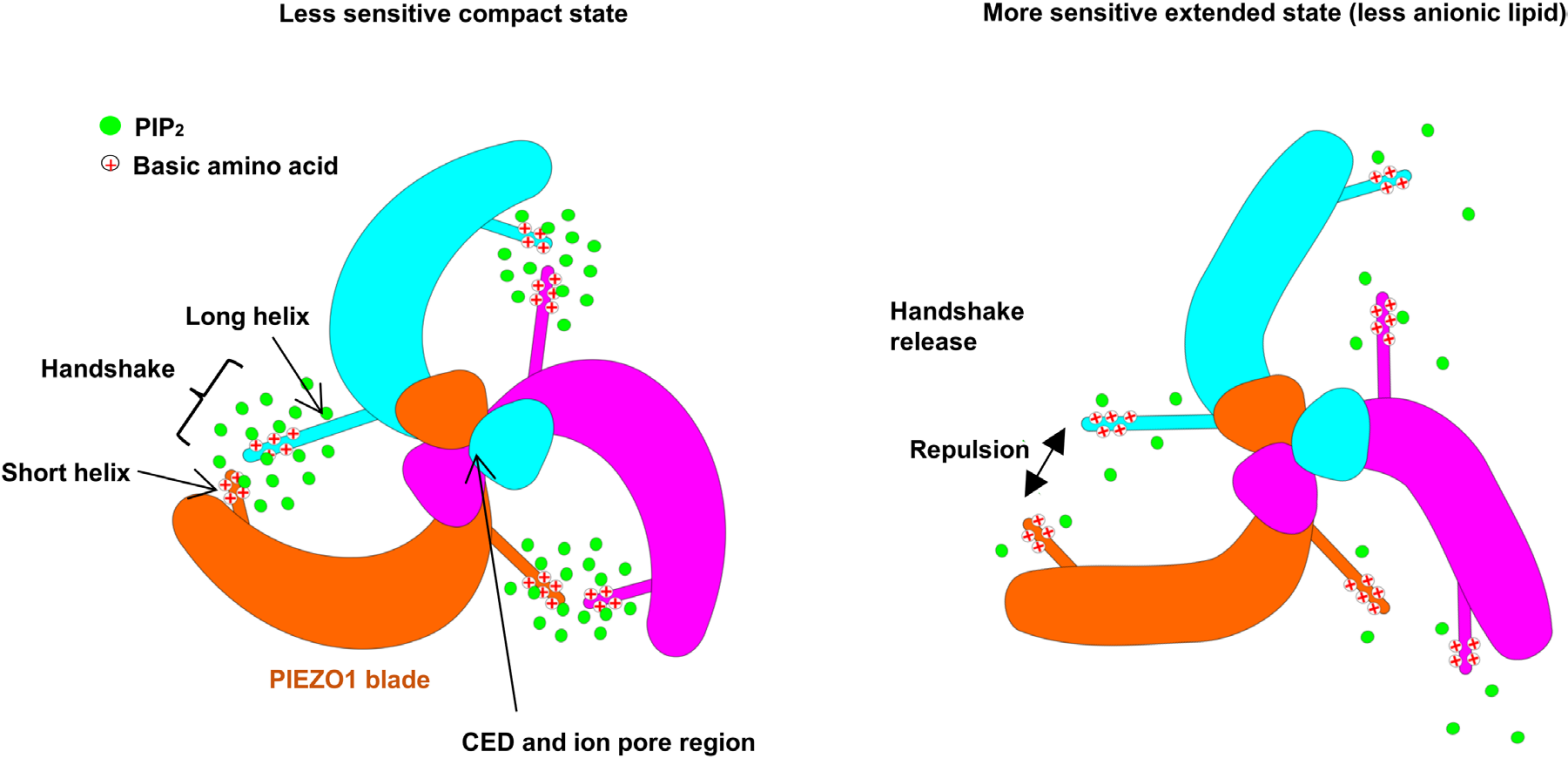
Summary of the handshake concept. An interblade handshake interaction regulates the dynamic motion of the blade domains and the depth of the membrane footprint. This interaction is facilitated by PIP_2_ lipids (green) which interact with basic amino acid residues (red crosses) in the handshake helices, allowing them to come together. Stochastic or regulated dispersion of PIP_2_ leads to loss of neutralization of the positive charge, leading to repulsion of the helices (handshake release), driving PIEZO1 to more extended states that are less compact and the ion pore region is more dilated (pore not shown). These states require less tension in the membrane to move to a flat active state. The C-terminal extracellular domain (CED) and the PIEZO1 ion pore region are also shown.

We suggest the existence of an interblade handshake interaction that functions to regulate the dynamic motion of the blade structures of PIEZO channels and thereby the sensitivity of the channels to pressure and (pressure-dependent) chemical agonist. We suggest that this interaction is enabled by anionic (negatively charged) lipids (e.g., PIP_2_ molecules) that interact with basic (positively charged) amino acid residues (e.g., arginines) in key blade helices, thereby allowing adjacent blades to “handshake” and make a more compact overall channel structure with a deeper membrane dome (Fig. 7). Compact channel structures will likely require more membrane tension to transition to the flat active state. The handshake concept also suggests that PIEZO1 blades function independently to adopt varying conformations. The initial asymmetry in the channel structures is conserved, resulting in asymmetry of the pore structures. Such a molecular mechanism could enable context-dependent differences in PIEZO sensitivities to mechanical and chemical activation, determined by local membrane lipid compositions that vary in different cell types and conditions, such as different lipid environments.

Our results suggest that in its closed state, the PIEZO channel is dynamic and transitions between multiple compact and extended nonsymmetric substates depending on the number of handshakes formed. This movement may be functionally relevant as force in the membrane is unlikely to be evenly distributed among the three PIEZO subunits (*11*). Nanoscopic fluorescence imaging studies have shown that PIEZO blades are dynamic in a membrane environment with blades being able to adopt different conformations at the resting state (*18*). The same studies showed that the blade conformational changes are asymmetric, in good agreement with our models. Structural studies have also highlighted the conformational flexibility of the blade domains, with structures solved which suggest asymmetric dynamic motion (*10*).

Our study suggests critical roles of lipids such as PIP_2_ in regulating hPIEZO1 and potentially other PIEZO conformational dynamics in the closed state. PIP_2_ is an essential membrane lipid with roles throughout cell biology including cytoskeletal dynamics, intracellular trafficking, and signal transduction both as a second messenger and by direct regulation of ion channels (*27*). PIP_2_ is a key player in mechanobiology, regulating the activity of other mechanosensitive ion channels such as KCNK2 (TREK-1) (*28*). Binding of anionic lipids to HCN2 channels acts like a key, unlocking the channel to allow activation (*29*). While phosphatidyl serine did not stabilize the handshake interaction to the same extent as PIP_2_ in our models, handshaking still occurred. It is possible that other anionic lipids, not included in our systems, also play a role in handshaking, allowing the cell to fine-tune the stability of handshaking via the number and type of anionic lipids present.

Receptor-mediated inhibition of PIEZO1 channels has been suggested to occur via PIP_2_ depletion (*30*). However, receptor-evoked hydrolysis of PIP_2_ also generates diacylglycerol and activates protein kinase C. In the same study, protein kinase C inhibited PIEZO1 channels, suggesting that receptor-mediated PIEZO1 inhibition could have occurred via protein kinase C rather than PIP_2_ depletion (*30*). Application of exogenous natural PIP_2_ appeared to rescue channel activity that had run down but the effects were inconsistent (*30*). We do not, however, exclude additional roles of PIP_2_ beyond handshaking. Numerous potential PIP_2_ interactions are seen in molecular dynamics (MD) simulations (Fig. 2D and fig. S5).

Calculations assuming a symmetrical PIEZO1 estimated a radius of curvature of ~40 nm in an asymptotically planar membrane under no tension (*31*). A radius of this type could not be obtained from our models because of channel asymmetry and a nonuniform dome, but we calculated instead an in-plane dome radius (*r*_b_), which decreased as expected with increasing handshakes (fig. S3C). We estimate that a radius of curvature of ~30 nm is equivalent to an *r*_b_ of ~11.5 nm, and thus our data (*r*_b_ > 12.0 nm) are in close agreement with the calculations of Haselwandter *et al.* (*31*, *32*).

It is also important to consider possible limitations of the simulations used in this study. We used CG-MD simulations for parts of this study which rely on approximations for the protein and lipid models. It has been suggested that Martini 2 may result in the overstabilization of protein-protein interactions and irreversible aggregation of proteins (*33*) particularly when predicting dimerization (*34*). While there are limitations to using Martini 2 to assess protein-protein interactions compared to newer versions of Martini forcefield, e.g., Martini 3, the handshake interaction we describe is a dynamic transient interaction that is unlikely to be an artifact caused by the overstabilization of protein-protein interactions. In addition, the handshake interactions are largely interfaced with lipid interactions, and we have been able to disrupt such interactions by mutation of lipid sites at the coarse-grained resolution and validate the results in laboratory experiments. Our AT simulations also partly address the limitation. The handshake helices, which have not been fully resolved in experimental structures, retain their helical structure in our AT simulations (fig. S25). The handshake interaction still occurs in our AT simulations; however, the timescales that are accessible were not sufficient to observe the full conformational ensemble. Therefore, to study handshaking and nonhandshaking states in AT resolution, we simulated WT hPIEZO1 from a compact and an extended starting structure (figs. S26 and S27). These showed PIP_2_ interactions consistent with those observed at coarse-grained resolution (fig. S28).

In summary, we suggest a mechanism that contributes to understanding of how a relatively small number of PIEZOs (two in human) serve many diverse roles across cell and organ types; spanning (for example) blood pressure regulation, lymphatic drainage, respiration, intestinal motility, immunity and sexual behavior in mammals, and root growth in plants. The handshake mechanism may have gone unrecognized previously because of its dynamic nature and the involvement of lipids, making it challenging to observe using laboratory structural biology techniques. With increasing availability of protein structures, there has been a shift toward understanding the relationship between structure and function. However, dynamic regions of protein structures are often overlooked because of being unresolved by structural techniques, and the details of lipid interactions are mostly missing. Our work reveals the idea of lipid-dependent protein-protein handshake regulating protein function. Increasing application of MD simulations to membrane proteins may reveal wider relevance of such a mechanism. Hence, our findings offer a different perspective on how PIEZOs and potentially other membrane proteins regulate their conformational dynamics.

## MATERIALS AND METHODS

### Molecular modeling of hPIEZO1 channel structure

Structural data corresponding to resolved elements of the full-length mPIEZO1 channel (*12*) were used as a structural template for the hPIEZO1 channel in combination with secondary structure prediction carried out using MEMSAT-SVM and PSIPRED (*35*, *36*) webserver tools. The final model was constructed on the basis of the combination of identified sequence conservation with mPIEZO1 and the output of the secondary structure prediction. The resulting full-length hPIEZO1 model was produced using MODELLER (v9.20) (*37*–*39*) for a single chain of hPIEZO1, which was then used to model the other chains with GROMACS (*40*) package tool gmx confrms. The final model was then assembled, and the energy was minimized. The trimeric structure of our full-length hPIEZO1 model shows good agreement with the cryo-EM structures of mPIEZO1 and mPIEZO2 (fig. S1, A and B). In addition, the PIEZO2 structure resolves the most of the transmembrane blade domains, which agree with our model.

The first ~4 turns of the handshake LH are resolved as helical in experimentally solved structures of mPIEZO1 (see also fig. S1A) (*9*–*11*) and mPIEZO2 (*20*). In the structure of mPIEZO2 [Protein Data Bank (PDB): 6KG7], the handshake SH is also partially resolved, with only the final ~2 turns of the helix unresolved and the structure and alignment of both the SH and LH are consistent with our full-length hPIEZO1 model (fig. S1, A and B). Secondary structure prediction also suggests the unresolved regions of SH and LH are α-helical in PIEZO1, and the positively charged nature of these regions is largely conserved (fig. S1, B and C).

The hPIEZO1 SHM model was generated using MODELLER (v9.20) (*37*–*39*). The mutations included in the model are as follows: SHM: R180A, R181A, R183A, R187A, and R189A (in each chain).

### CG-MD simulations

The full-length hPIEZO1 models were converted to a coarse-grained resolution using the martinize script (*41*). An elastic network model with a cutoff distance of 7 Å was used to model the protein secondary and tertiary structure. The elastic force constant was 1000 kJ/mol. The CG-MD simulations were run using the Martini 2.2 (*41*) forcefield and GROMACS 5.0.2 (*40*). The hPIEZO1 models were inserted into a complex asymmetric bilayer using the INSert membrANE tool (*42*) containing approximately 5900 lipids with lipid composition selected to mimic that of the endothelial membrane (*21*, *22*). The membrane compositions are described in Table 1. The system with a composition containing 5% PIP_2_ was chosen to mimic the physiological endothelial membrane. An additional system was set up containing 0% PIP_2_, which were replaced with POPS molecules. All other lipid concentrations were the same. The systems were assembled in a box of dimensions 44 nm by 44 nm by 24 nm. The systems were neutralized with 150 mM NaCl. The models were then energy minimized for 5000 minimization steps. The systems were subsequently equilibrated with protein particles restrained (1000 kJ mol^−1^ nm^−2^) for 100 ns to allow the bilayer to equilibrate around the hPIEZO1 model and adopt the dome shape previously shown (*9*). All simulations were performed at 323 K, with protein, lipids, and solvent separately coupled to an external bath using the V-rescale thermostat (*43*) (coupling constant of 1.0) with semi-isotropic conditions and compressibility of 3 × 10^−6^ using the Berendsen barostat (*44*) during equilibration and the Parrinello-Rahman barostat (*45*) during production simulations. Lennard-Jones and Coulombic interactions were shifted to zero between 9 and 12 Å and between 0 and 12 Å, respectively. Following equilibration, a maximum of 10 lipid molecules (<0.17% of total lipid content) were removed that flipped between leaflets to restore membrane asymmetry. After this step, the systems were further equilibrated. Removal of lipids did not affect box dimensions, indicating lack of effect on membrane topology (fig. S2). Following equilibration, lipids are free to move and flip between leaflets. For each hPIEZO1 model, five unrestrained repeat simulations were run for 3 μs (or 5 μs where stated) using an integration step of 20 fs.

### CG-MD analysis

All analysis was performed using GROMACS (*40*) (5.0.7) (gmx mindist, gmx trjconv, gmx densmap, gmx dist, and gmx angle), PyMOL (alignment), and inhouse scripts. GROMACS analysis was performed on concatenated (gmx trjcat) and centered (gmx trjconv) trajectories, unless otherwise stated. The electrostatic profile of PIEZO1 was calculated using PDB2PQR followed by VMD APBS Electrostatics (*46*).

#### Handshake analysis

The distance between residue 176 of the handshake SH and residue 1912 of the handshake LH of the clockwise neighboring chain was calculated using gmx dist for each pair of helices. A distance of less than or equal to 1.5 nm was defined as a handshake interaction. The number of handshakes formed in each frame of the simulation was calculated using NumPy (*47*). Histogram of handshake helix distances was calculated and plotted using Matplotlib (*48*). Graphs of handshake conformation frequency were plotted using Matplotlib (*48*).

#### Blade dynamic analysis

Frames were extracted corresponding to conformations associated with 0, 1, 2, and 3 handshake interactions, respectively. The effect of the handshake interactions on the dynamic nature of the blade domains was quantified using two measurements for the conformation of the blade. The distance between the center of geometry of the N-terminal THU (THU1) and the central pore was measured using gmx dist. This provides a measure of how extended the blades are. The angle formed by the vectors defined by the center of geometry of THU1 to THU6 and THU6 to THU9 was measured using the gmx angle. This provides a measurement of how curved the blade domain is in the *xy* plane. 2D histograms were calculated using NumPy (*47*) and plotted using Matplotlib (*48*). The data were normalized for the number of frames extracted corresponding to each conformation and for the bin size.

#### Protein contact analysis

Protein contact analysis was performed using gmx mindist in GROMACS (*40*) 5.0.7 to localize the region of interaction between the two handshake helices. Contacts between the residues of the SH (residues 176 to 203) and the LH (residues 1912 to 1951) were calculated using gmx mindist for each chain. The data were normalized for the number of frames and averaged across the three monomers. Data were plotted using Matplotlib (*48*).

#### Lipid contact analysis

Contacts between the CG lipid head groups and protein residues were calculated using gmx mindist for each lipid group included in the membrane system. The data were then averaged across the five repeat simulations and normalized for the number of frames and number of lipids in the system. The number of contacts was then averaged across the three monomers which form the hPIEZO1 channel. Data were plotted using Grace (https://plasma-gate.weizmann.ac.il/Grace/).

#### Lipid density analysis

Lipid density was calculated using GROMACS (*40*) 5.0.7 (gmx densmap). The simulation trajectory files were fitted to the hPIEZO1 coordinates of the first frame of the simulations using gmx trjconv. The fitted simulation data of each repeat simulation were concatenated such that the calculated lipid density was representative of the lipid localization across all five simulations. The 2D density map was colored using gimp (https://gimp.org).

#### Membrane depth analysis

The depth and topology of the hPIEZO1 membrane footprint for each system were determined using a Python script described in (*12*). The simulation trajectory was fitted to the protein coordinates, and the coordinates of the CG phosphate head group beads in each frame were extracted into separate files using GROMACS (*40*) (gmx trjconv). This generated a height map of the bilayer leaflets corresponding to the average membrane depth across all simulation frames. The depth and topology of the hPIEZO1 membrane footprint for the extended and compact structures of WT hPIEZO1 in an endothelial model membrane containing 5% PIP_2_ were calculated for the frames extracted in which all three chains formed a compact conformation (in which three handshake interactions are formed) and for the frames extracted in which all three chains formed an extended conformation (in which the blade distance from pore > 13 nm for all three chains).

#### *Projected area and projected radius (r*_b_) *analysis*

The projected area of the hPIEZO1 channel was calculated as described in (*15*). Gmx dist was used to calculate the distances between the Cα of L71, located within the THU at the N terminus, of each chain. The distance was then used to calculate the radius and projected area as described in (*15*).

#### Lipid pool analysis

The number of PIP_2_ lipids within 2 nm of the SH of the handshake interaction was calculated using gmx mindist for the lipid head group with a cutoff of 2 nm.

### AT tension simulations

A snapshot from CG simulations was selected for WT hPIEZO1 (in compact and extended conformations) and SHM PIEZO1 backmapped to an AT resolution as described in (*49*). The box size was increased by 40% in the *z* dimension to withstand applied tension following conversion to AT resolution. PIEZO1 is Ca^2+^ permeable, so 3 mM Ca^2+^ ions (50 ions) was added to the box and neutralized with counterions. The obtained system was energy minimized and equilibrated in three protein restrained (1000 kJ mol^−1^ nm^−2^) NPT ensemble runs of 20,000 steps, each with an increasing time step from 0.2 to 2 fs. A further equilibration step of 5 ns with a time step of 2 fs with protein particles restrained (1000 kJ mol^−1^ nm^−2^) was performed. All AT systems were simulated using GROMACS 2020 (*40*) with CHARMM36 (*50*) forcefield and a 2-fs time step. A Berendsen semi-isotropic pressure coupling (*44*) at 1 bar was used during all the equilibration phases. The Parrinello-Rahman barostat (*43*) was used for the tension simulations. The simulations were performed at 323 K. Long-range electrostatics were managed using the particle-mesh Ewald method. Bond lengths were constrained using the LINCS algorithm. Tension simulations were performed using 50-ns unrestrained simulations in which pressure was applied to the bilayer plane (*xy* plane) at −30 bar (64.193 mN/m) and +1 bar (0 mN/m). The pressure in the *z* dimension was kept at +1 bar. The tension used in our simulations is higher than physiologically relevant values but was used to facilitate the shorter timescales accessible for AT simulations.

The surface tension (γ) was calculated using the equation

$$\gamma =L_{z}\left(p_{N}-p_{L}\right)$$

where *L*_z_ equals the box size in the *z* axis, *p*_N_ equals the pressure in the *z* axis (*p*_zz_), and *p*_L_ equals (*p*_xx_ + *p*_yy_)/2.

### AT simulation analysis

All analysis was performed using GROMACS (*40*) (2020.3) (gmx mindist, gmx trjconv, and gmx dist), PyMOL (alignment), and inhouse scripts. GROMACS analysis was performed on concatenated (gmx trjcat) and centered (gmx trjconv) trajectories, unless otherwise stated. Pore volumes were calculated using trj_cavity (*51*) and GROMACS (*40*) (5.1.4). Handshake, lipid contacts, and projected area analyses were performed as described for CG simulations but using GROMACS 2020.3.

#### Blade height analysis

The height of the PIEZO1 blades was calculated by measuring the distance between the center of geometry of the N-terminal THU (THU1) and the central pore in the *z* dimension using gmx dist.

#### Volume of pore

The volume of the central pore in the final snapshot of each simulation was calculated using the tool trj_cavity (*51*). The considered residues were 2433 to 2462 in each chain. Options dim 3 (i.e., degree of buriedness) and spacing 1.4 Å were used.

#### Dimensions of pore

The dimensions of the pore inner cavity were calculated at the top (Y2444), middle (V2450), and bottom (F2454) of the pore inner helix TM38. In the SHM channel, V2450 of chain C is rotated by ~45°C such that L2449 lines the inner pore (fig. S10). To calculate the dimensions of the pore at this level, we, therefore, used V2450-V2450-L2449 as the residues for this calculation in the SHM channel. The distance between the residues in each chain was calculated using gmx dist. These values were then used to calculate the area of the triangle formed by the three residues using Heron’s formula

Area=√s(s−a)(s−b)(s−c)

where *s* refers to the semiperimeter of a triangle with sides of lengths *a*, *b*, and *c*.

### DNA constructs and cloning

WT and SHM hPIEZO1 were subcloned from the hPIEZO1-mTurquoise-pcDNA6 construct (*52*). Overlapping PIEZO1 and pcDNA6 fragments were generated by polymerase chain reaction (PCR) using PrimeSTAR HS DNA Polymerase (TaKaRa), and PCR primers are shown in Table 2. Fragments were assembled using GeneArt Gibson Assembly cloning (Invitrogen).

**Table 2.** PCR primers.

Reaction	Primers
WT hPIEZO1 fragment	Forward: ACTTAAGCTTATGGAGCCGCACGTGCTC; reverse: CGCCACTGTGCTGGATATCTGCACTCCTTCTCACGAGTCCACTTGATCATGGT
WT pcDNA6 fragment	Forward: TGCGAAAGCAGCGGCCAGCGCTGATGCCGCTGTAGGGGCCAGCGTTGC; reverse: GCGGCTCCATAAGCTTAAGTTTAAACGCTAGC
SHM hPIEZO1 fragment	Forward: GCGGCATCAGCGCTGGCCGCTGCTTTCGCAGTCACGGCCCACTGGC; reverse: CGCCACTGTGCTGGATATCTGCACTCCTTCTCACGAGTCCACTTGATCATGGT
SHM pcDNA6 fragment	Forward: TGCGAAAGCAGCGGCCAGCGCTGATGCCGCTGTAGGGGCCAGCGTTGC; reverse: ACCATGATCAAGTGGACTCGTGAGAAGGAGTGCAGATATCCAGCACAGTGGCG

### Cell lines

The HEK293 cell line was maintained at 37°C, 5% CO_2_, 95% air atmosphere in Dulbecco’s modified Eagle’s media supplemented with 10% fetal calf serum, 2 mM l-glutamine, penicillin (100 U ml^−1^), and streptomycin (100 μg ml^−1^). Stable cell lines overexpressing hPIEZO1 WT or SHM were generated by transient transfection of HEK293 cells using Lipofectamine 3000. Forty-eight hours posttransfection, selection antibiotic blasticidin (5 μg ml^−1^) was added.

### Electrophysiology

HEK293 cells stably expressing WT or SHM hPIEZO1 were seeded into a T25 tissue culture flask. Cells were detached using trypsin and seeded onto poly-d-lysine (Thermo Fisher Scientific)–coated coverslips. In response to mechanical stimulation, ionic currents through outside-out membrane patches were recorded using the standard patch-clamp technique in voltage-clamp mode at a holding potential of −80 mV. Patch pipettes were fire polished and had a resistance of 4 to 8 megohm when filled with the solution of the following composition: 140 mM NaCl, 10 mM Hepes, and 5 mM EGTA, titrated to pH 7.4 using NaOH. The same solution was used as a bath solution. For PIP_2_ depletion, cells were preincubated in bath solution containing 50 μM wortmannin (Cayman Chemical, Ann Arbor, USA) for 90 min with two ~30-s applications of 100 μM ATP (Sigma-Aldrich, Poole, UK) separated by 30 min before subsequent patch-clamp recording with 50 μM wortmannin in the patch pipette solution. For diC8 PIP_2_ studies, 10 μM diC8 PIP_2_ [1-(1,2R-dioctanoylphosphatidyl)inositol-4,5-bisphosphate trisodium salt from Cayman Chemical, Ann Arbor, USA] was included in the patch pipette solution. A pressure protocol was applied to the patch pipette increasing from 0 to 120 mmHg (Δ15 mmHg) using the High Speed Pressure Clamp HSPC-2-SB System. Pressure steps were applied for 200 ms followed by an interval of 20 s. All recordings were made using an Axopatch-200B amplifier (Axon Instruments Inc., USA), Digidata 1550B, and pClamp 10.7 software (Molecular Devices, USA) at room temperature. Current records were filtered at 2 or 5 kHz and acquired at 5 or 20 kHz. All experiments were randomized and blinded.

### Intracellular Ca^2+^ measurement

HEK293 cells stably expressing WT or SHM hPIEZO1 or UT were plated in a 96-well plate 24 hours before recording at a density of 5 × 10^4^ cells per well. Cells were treated in standard bath solution (SBS) (composition 135 mM NaCl, 5 mM KCl, 1.5 mM CaCl_2_, 1.2 mM MgCl_2_, 8 mM glucose, and 10 mM Hepes, titrated to pH 7.4 with NaOH) containing 2 μM Fura-2, acetoxymethyl (AM) ester and 0.01% pluronic acid for 1 hour at 37°C. Cells were then washed with SBS for 30 min at room temperature. Measurements were made in SBS containing 0.01% pluronic acid and 0.3% dimethyl sulfoxide (DMSO), at room temperature on a FlexStation 96-well plate reader. Cells were treated with increasing concentrations of Yoda2 (*25*) (0.03, 0.1, 0.3, 1, 3, 10, and 30 μM) to generate a dose-response curve. Yoda2 (*25*) was prepared in stocks of concentration 20 mM in DMSO. For PIP_2_ depletion, the protocol was the same as for the electrophysiology except including Fura-2-AM incubation.

### Western blot

Cells were lysed in NP-40 cell lysis buffer (Thermo Fisher Scientific), supplemented with protease inhibitor cocktail (Sigma-Aldrich) and phosphatase inhibitor (Millipore). Samples were centrifuged at 4°C for 10 min at 12,000*g*, and the supernatant was collected. Following protein quantification with bicinchoninic Acid (BCA) assay according to manufacturer’s instructions (Thermo Fisher Scientific, Rapid Gold BCA Protein Assay Kit), 15 μg of protein in Laemmli reducing buffer was heated at 37°C for 30 min. Samples were loaded on precast 7.5% polyacrylamide gradient gel (Bio-Rad) and subjected to electrophoresis. Proteins were then transferred onto a polyvinylidene difluoride membrane using wet transfer for 3 hours at 400 mA. After blocking in 5% milk–Tris-buffered saline with 0.1% Tween 20 detergent (TBS-T), membranes were incubated with anti-PIEZO1 antibody (Invitrogen, MA5-32876) overnight at 4°C. Membranes were washed and incubated with secondary antibody anti-mouse horseradish peroxidase (HRP) for 1 hour at room temperature, washed, and imaged using the SuperSignal West Dura ECL (Thermo Fisher Scientific) and the IBright (Thermo Fisher Scientific) imaging system. Membranes were stripped, blocked, and incubated with an anti-HSP90 (SantaCruz, sc-13119) antibody overnight. Following mouse-HRP secondary antibody (715-035-150-JIR) incubation as previously described, membranes were imaged. Densities were analyzed using ImageJ.

### Patch-clamp and Ca^2+^ measurement analysis and statistics

UT, WT, and SHM cells were randomized and blinded to the experimenter and analyst until all experiments were complete. Patch-clamp data were analyzed and plotted using pClamp 10.6 and MicroCal Origin 2018 (OriginLab Corporation, USA) software. Data from patches that ruptured at or before the 120-mmHg pulse were excluded. In PIP_2_ depletion conditions without diC8 PIP_2_, patches were more vulnerable to disruption, and data could often only be obtained up to 60 mmHg. Pressure curves were constructed in Origin and fitted with the Boltzmann function *I* = *I*_min_ + (*I*_max_ − *I*_min_)/{1 + exp [(*p* − *p*_50_)/d*p*]}, where *I*_min_ and *I*_max_ are the minimum and maximum current, *p*_50_ is the mid-point, and d*p* is the Boltzmann coefficient. Inactivating currents were fitted with the exponential function *y* = A1 × exp(−*x*/τ) + *y*0, where A1 is the current at time zero, τ is the time constant, and *y*0 is the residual (noninactivating) current. For WT and SHM Ca^2+^ data, mean intensity values from UT cells for each matched experiment were treated as background and subtracted. Fura-2 fluorescence ratios (*R*) values were fitted in Origin with the Hill function *R* = *R*_min_ + (*R*_max_ − *R*_min_) × ([Yoda2]^HC^/EC_50_^HC^ + [Yoda2]^HC^), where *R*_min_ and *R*_max_ are the minimum and maximum ratio, [Yoda2] is the Yoda2 concentration, EC_50_ is the mid-point, and HC is the Hill coefficient.

### Molecular modeling of mPIEZO2 handshake helix structure

Structural data corresponding to the resolved elements of the full-length mouse mPIEZO2 (*20*) were used as a structural template for the mPIEZO2 + handshake helix model. The regions corresponding to the handshake helices were unresolved by cryo-EM. The equivalent regions were identified by sequence alignment with mPIEZO1, and secondary structure prediction was carried out using MEMSAT-SVM and PSIPRED (*35*, *36*) webserver tools. The results of which suggested that the unresolved residues form the α helices. The final target-template alignment was constructed using the resolved mPIEZO2 sequence plus the identified handshake helices. The resulting mPIEZO2 + handshake helix model was produced using MODELLER (v9.20) (*37*–*39*) for a single chain of mPIEZO2. This model was aligned to the coordinates of the other two chains using PyMOL. The final model was then assembled and energy minimized.

### Multiple sequence alignment of 15 PIEZO orthologs

PIEZO orthologs across eukaryotes were selected for multiple sequence alignment to look at the conservation of positive charge in the regions expected to form the handshake helices. Fifteen PIEZO orthologs were selected: hPIEZO1 (UniProt: Q92508), mPIEZO1 (UniProt: E9PUQ9), mPIEZO2 (UniProt: Q8CD54), dog (UniProt: A0A8I3MUY4), zebrafish (UniProt: A0A8N7TDV6), pig (UniProt: A0A480F0E9), tufted duck (UniProt: A0A6J3DPG2), bovine (UniProt: F1MD64), cat (UniProt: A0A337SVB0), koala (UniProt: A0A6P5KK49), western European hedgehog (UniProt: A0A1S3WLZ8), rhesus macaque (UniProt: A0A5F7ZT10), chicken (UniProt: A0A8V0Y724), Atlantic cod (UniProt: A0A8C4YZ71), and *Arabidopsis thaliana* (UniProt: F4IN58). Multiple sequence alignment was performed on 15 PIEZO orthologs using the Clustal Omega program (*53*). Figures were made using Jalview (*54*).
