# Supplementary material for: Regulation of PIEZO1 channel force sensitivity by interblade handshaking
Source: Sci Adv. 2025 Jun 13;11(24):eadt7046. doi: 10.1126/sciadv.adt7046 (PMC12164982; doi:10.1126/sciadv.adt7046)
Supplement: Supplementary file 1 — Figs. S1 to S28 Legend for movie S1 [file sciadv.adt7046_sm.pdf]

Science Advances
AAAS

Supplementary Materials for

Regulation of PIEZO1 channel force sensitivity by interblade handshaking

Katie A. Smith *et al.*

Corresponding author: Eulashini Chuntharpursat-Bon, medechu@leeds.ac.uk;
David J. Beech, d.j.beech@leeds.ac.uk; Antreas C. Kalli, a.kalli@leeds.ac.uk

Sci. Adv. **11**, eadt7046 (2025)
DOI: 10.1126/sciadv.adt7046

The PDF file includes:

Figs. S1 to S28
Legend for movie S1

Other Supplementary Material for this manuscript includes the following:

Movie S1

Figure S1

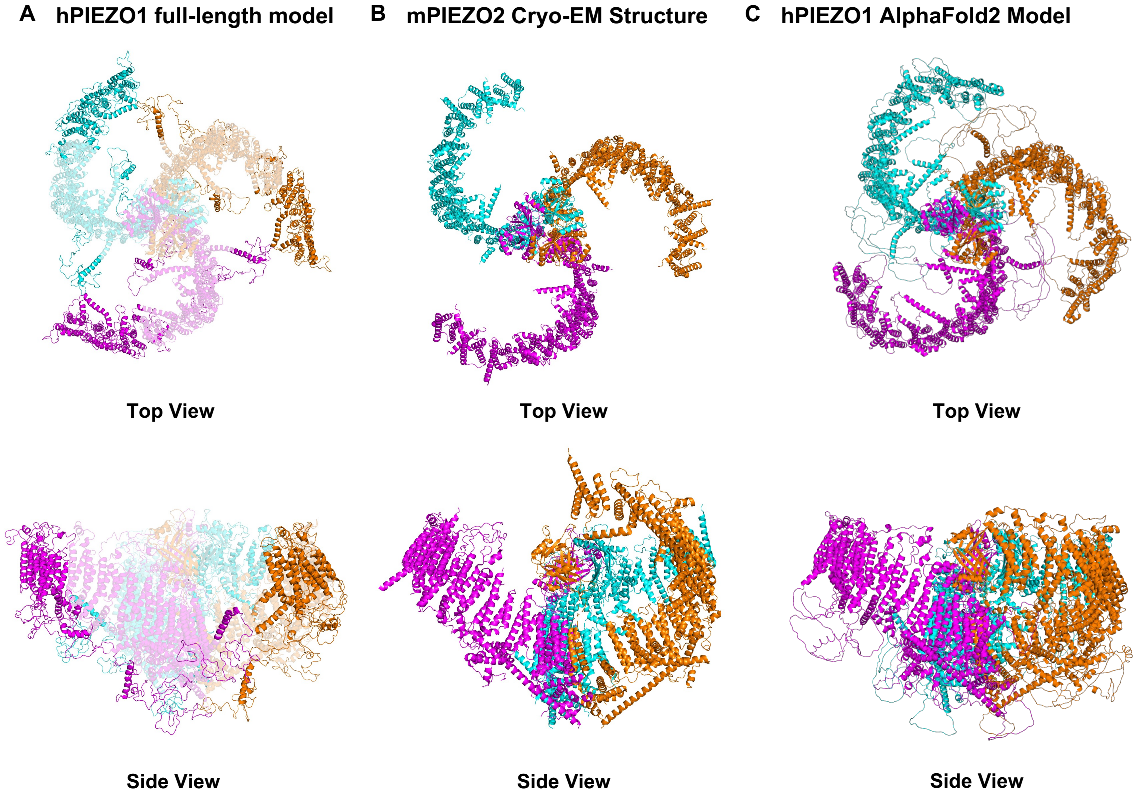

Figure S1: Full-length structural model of human PIEZO1 (hPIEZO1) compared to full-length mouse PIEZO2 (mPIEZO2) cryo-EM structure and hPIEZO1 AlphaFold2 model. (**A**) Full-length structural model of hPIEZO1 channel shown in a cartoon representation with chains (one chain per one hPIEZO1 protein) in orange, purple and cyan shown from above (top) and side (bottom). Regions resolved in cryo-EM studies of mouse PIEZO1 (mPIEZO1) channel are shown in light colour and unresolved regions modelled are shown in darker colour (see Methods). (**B**) mPIEZO2 cryo-EM structure (PDB: 6KG7). Shown in cartoon representation with chains in orange, purple and cyan shown from above (top) and side (bottom). (**C**) hPIEZO1 AlphaFold2 model (AF-Q92508-F1-v4). Shown in cartoon representation with chains in orange, purple and cyan shown from above (top) and side (bottom).

Figure S2

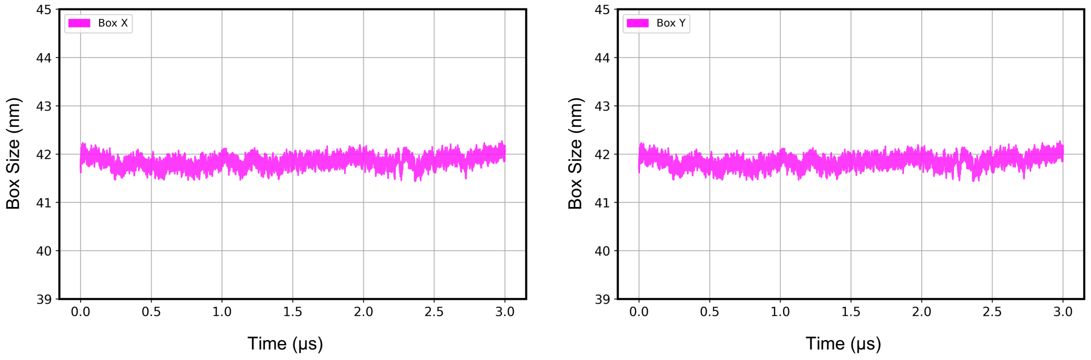

Figure S2: Calculation of box size in x and y axes. The size of the boxes in the x an y axes was calculated over simulation time following removal of flipped lipids. The box size in x and y corresponds to the dimensions of the bilayer in the x and y-axes. Stable box size in x and y-axes suggests a stable membrane conformation.

Figure S3

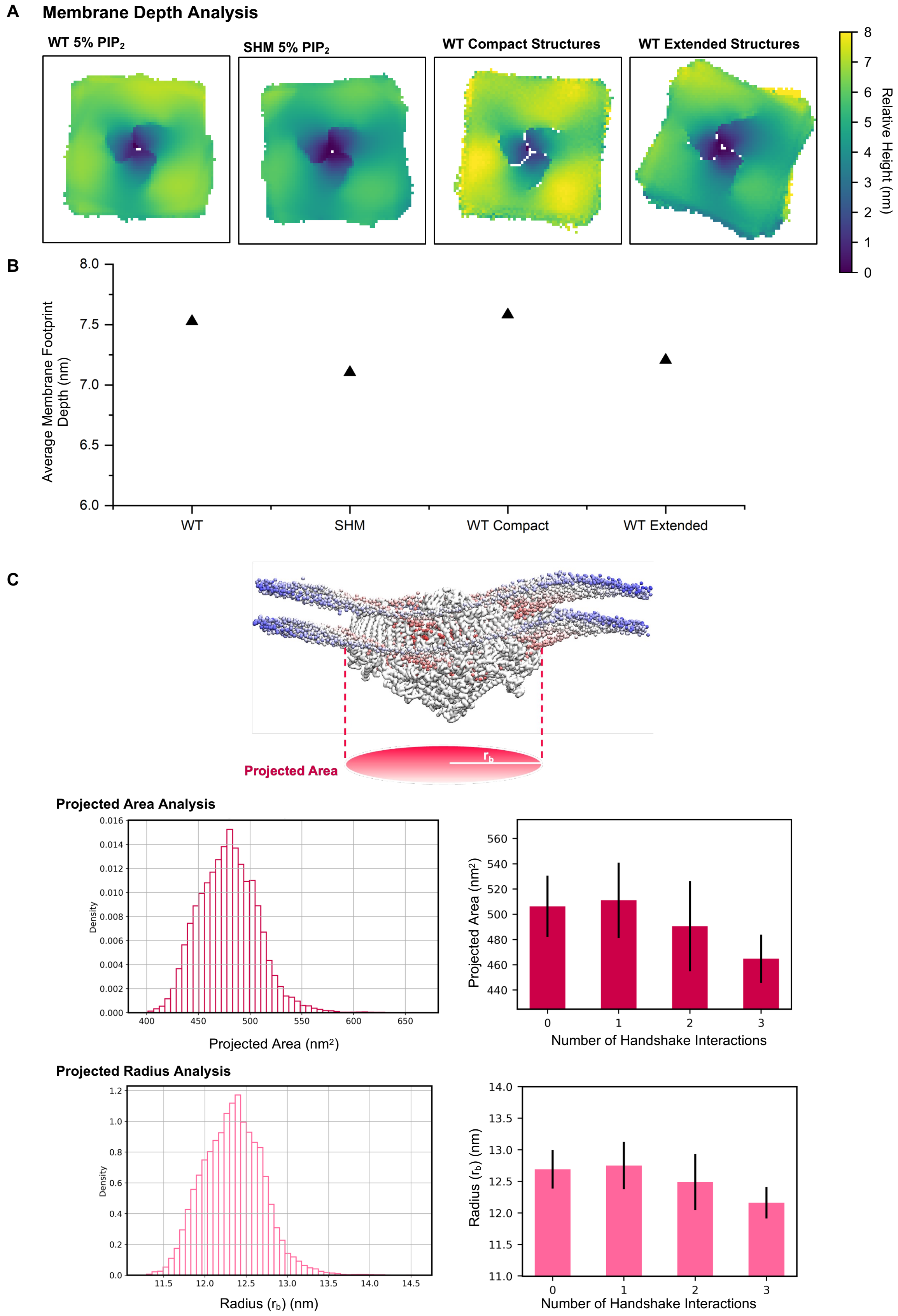

Figure S3: Membrane depth analysis. (**A**) Height map of coarse-grained phosphate beads of the upper leaflet calculated across all 5 repeat simulations of WT and SHM

hPIEZO1 simulated in an endothelial model membrane containing 5% PIP_2. Height maps for the WT compact and extended structures were calculated by averaging the relative height of the coarse-grained phosphate beads of the upper leaflet across all frames in which all 3 blades are compact or all 3 blades are extended. (**B**) Average depth of the upper leaflet of the membrane WT and SHM PIEZO1 systems containing 5% PIP_2 and frames in which WT PIEZO1 adopts compact and extended structures. (**C**) Projected area and projected radius analysis calculated for hPIEZO1 simulated in model endothelial membrane containing 5% PIP_2. Depiction of the projected radius (r_b) measurement (top). Projected area of the channel corresponds to the area of a circle defined by the end of the each blade, the projected radius (r_b) is the radius of this circle. Histograms of projected areas and r_b are shown (left, below). The average projected areas and r_b were calculated for frames in which hPIEZO1 forms 0, 1, 2 and 3 handshakes (right, below).

Figure S4

Protein Contacts Analysis between the Short Handshake Helix (blue) with the long handshake helix (pink)

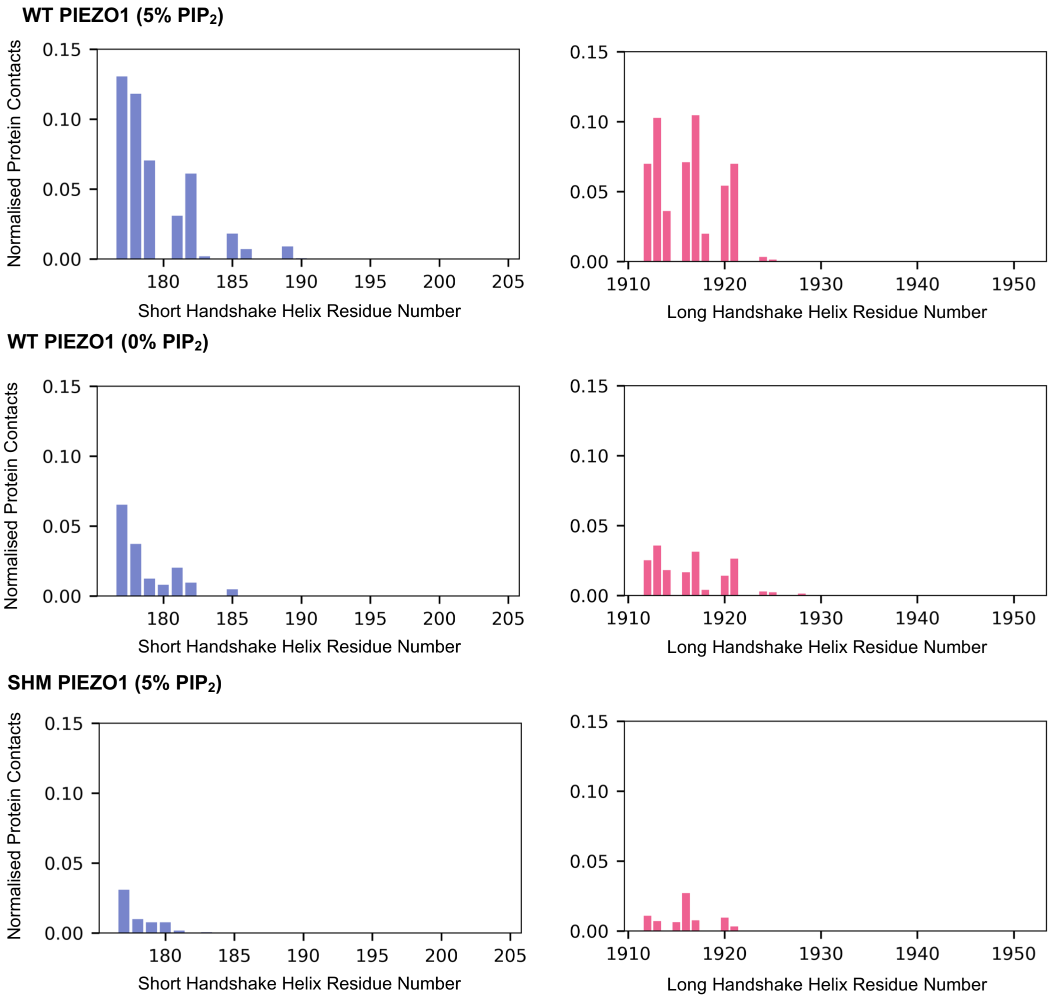

Figure S4: Protein contacts formed between the short helix (SH) and long helix (LH) of the handshake interaction. Protein contacts calculated between SH and LH which form the handshake interaction for WT hPIEZO1 simulated an endothelial model membrane containing 5% PIP_2 (top) and 0% PIP_2 (middle) and SHM hPIEZO1 simulated in an endothelial model membrane containing 5% PIP_2 (bottom). Protein contacts were calculated between residues 176-203 of SH and residues 1912-1951 of LH on the neighbouring chain. Contacts with SH (left) are shown in blue, and contacts with LH (right) are shown in pink. Contacts were averaged for the 3 chains and normalised for the number of frames.

Figure S5

Lipid Contacts

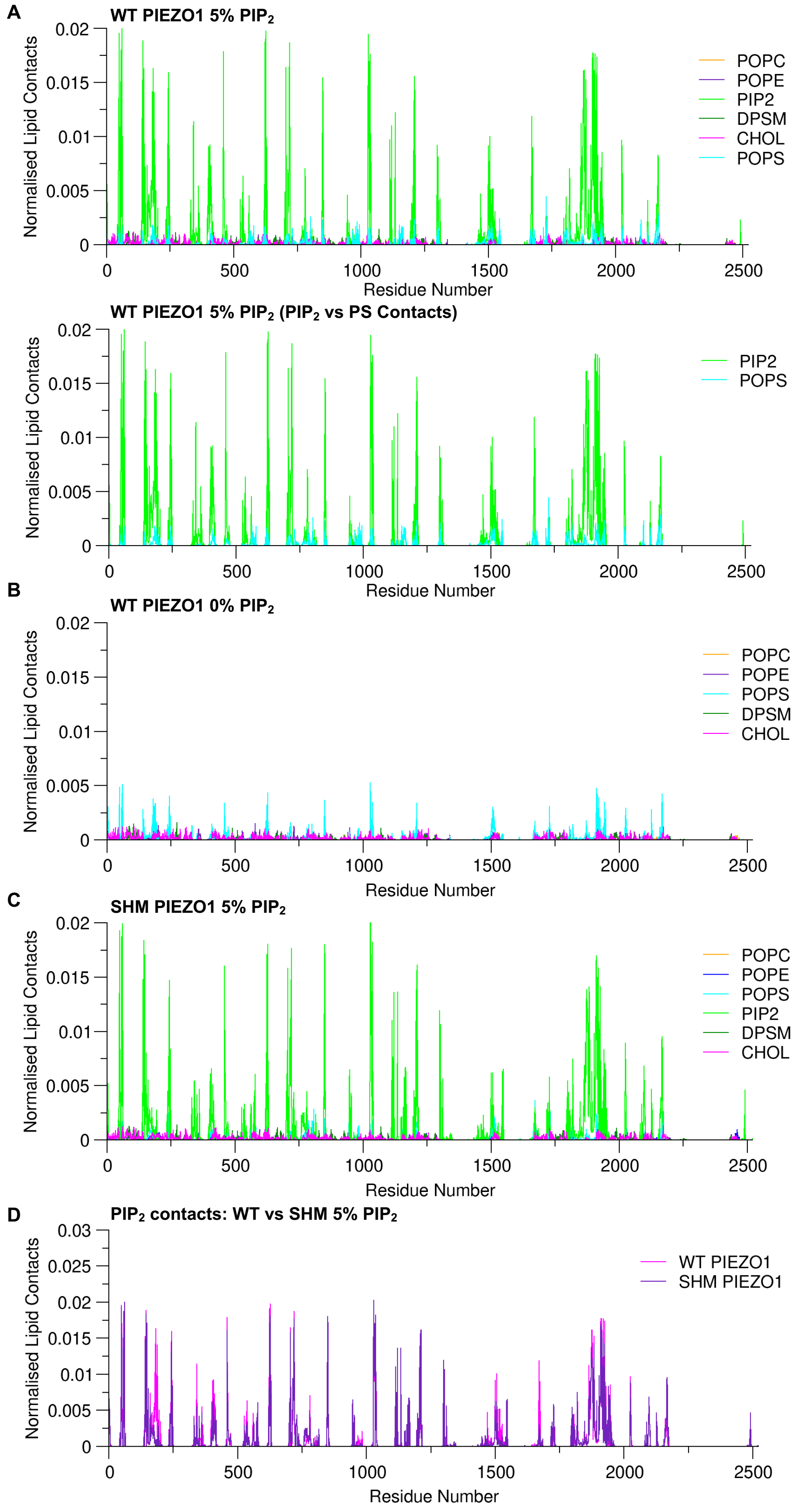

Figure S5: Lipid contacts analysis of hPIEZO1 residues with lipids present in model membranes. hPIEZO1 lipid contacts calculated for each lipid type included in the computer simulations. Lipid contacts were normalised for the number of frames and the number of lipids of each type. Lipid contacts were averaged across the 3 hPIEZO1 subunits. (**A-D**) POPC (orange), POPE (purple), POPS (cyan), PIP_2 (green), DPSM (dark green), CHOL (magenta). (**E**) Comparison of PIP_2 contacts with WT (magenta) and SHM PIEZO1 (purple) simulated in an endothelial model membrane containing 5% PIP_2.

Figure S6

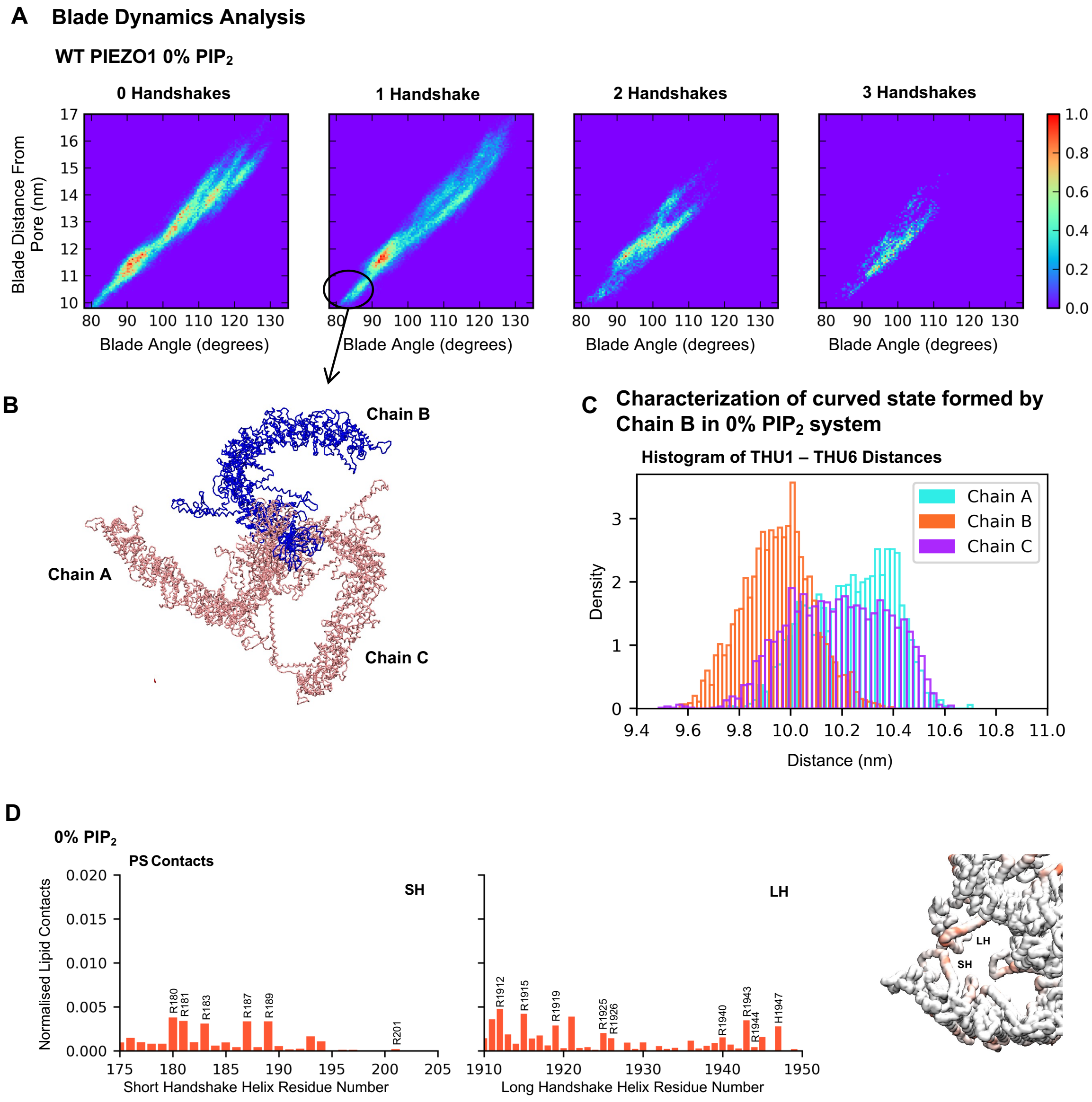

Figure S6: Analysis of blade dynamics for WT hPIEZO1 simulated in an endothelial model membrane in the absence of PIP_2 (**A**) Blade dynamics analysis for WT hPIEZO1 simulated in an endothelial model membrane containing 0% PIP_2. 2D histograms of the distance between the N-terminal THU and the pore and the angle formed by the blade. Histograms were calculated separately for frames in which hPIEZO1 forms 0, 1, 2 and 3 handshakes. (**B**) Frame extracted from simulation trajectory in which 1 chain forms a conformation in which the blade distance from pore < 11 nm and blade angle < 90. Chain B which forms this conformation is shown in blue, chain A and chain C are shown in pink. (**C**) Characterisation of more curved blade conformation formed by one of the chains in 0% PIP_2 system. Histogram of distances between THU1 and THU6 for Chain A (cyan), Chain B (orange) and Chain C (purple) of PIEZO1. Histogram was calculated for frames in which the blade distance from pore < 11 and blade angle < 90.

Figure S7

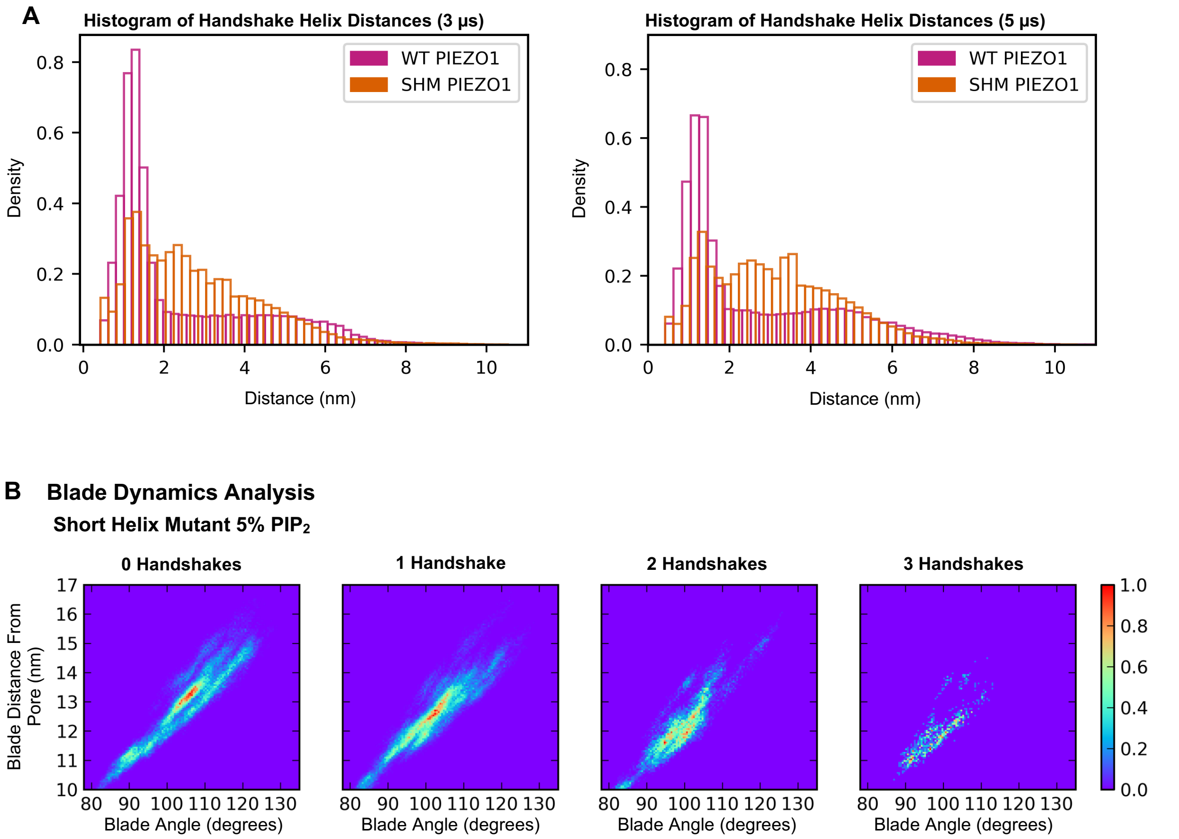

Figure S7: Analysis of interchain handshaking by SHM hPIEZO1. (**A**) Histogram of distances between the final residue of each helix which forms the handshake interaction for WT (pink) and SHM (orange) hPIEZO1 simulated in an endothelial model membrane containing 5% PIP_2 for 3 μs (left) and 5 μs (right). (**B**) Blade dynamics analysis for short helix mutant (SHM) hPIEZO1 simulated in a membrane containing 5% PIP_2. 2D histograms of the distance between the N-terminal THU and the pore and the angle formed by the blade. Histograms were calculated separately for frames in which hPIEZO1 forms 0, 1, 2 and 3 handshakes.

Figure S8

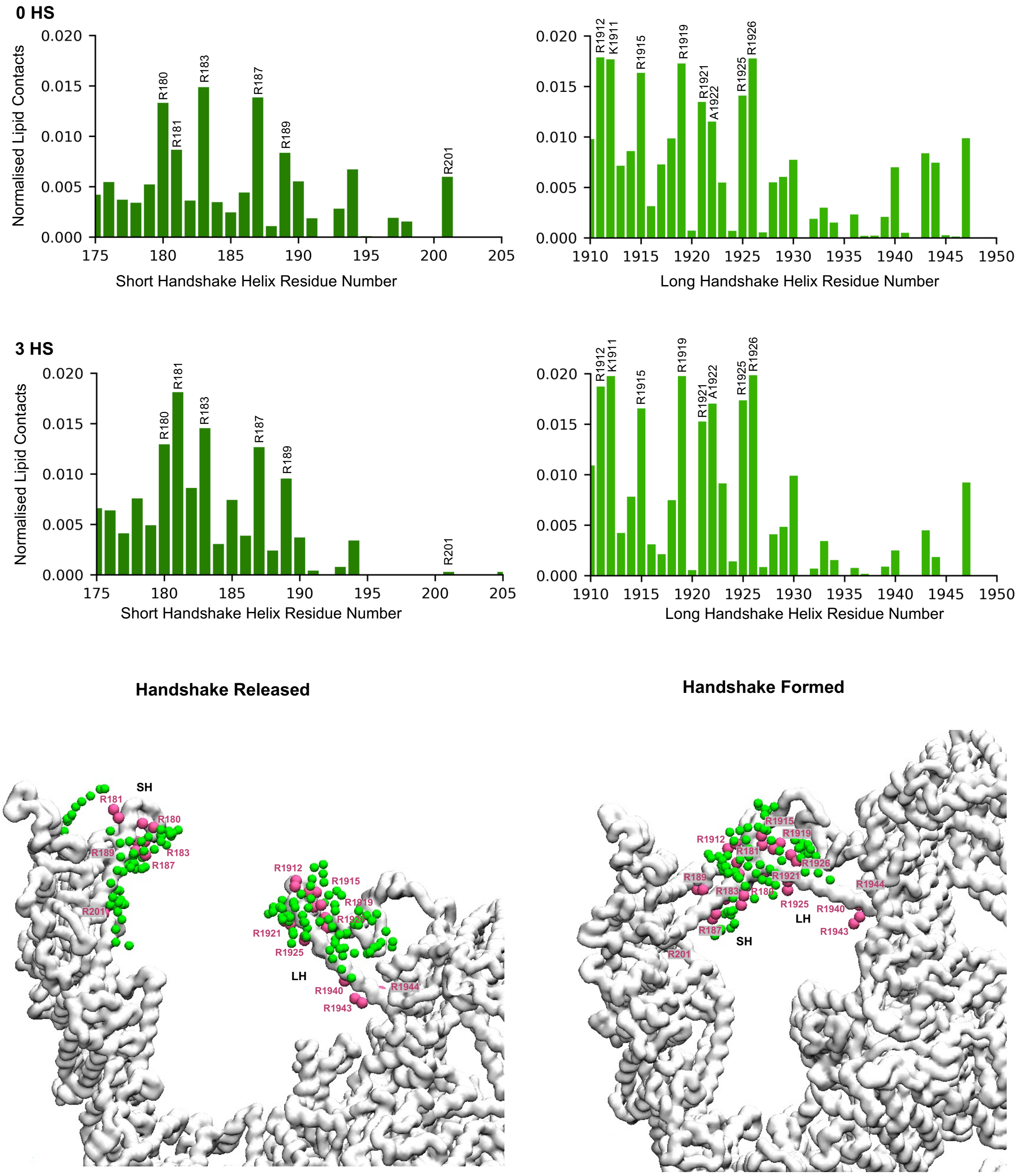

Figure S8: Lipid contacts analysis of WT hPIEZO1 handshake helix residues with PIP_2 lipids in all frames and frames where hPIEZO1 forms 0 and 3 handshakes. Lipid contacts between PIP_2 lipids and SH and LH of handshake interaction (top). Data corresponding to arginine residues are labelled. Lipid contacts were normalised for the number of frames and the number of PIP_2 lipids. Lipid contacts were averaged across the 3 hPIEZO1 subunits. Snapshots showing PIP_2 interactions with handshake helices when handshake is released (left) and formed (right). hPIEZO1 backbone is shown in surface representation in white. Arginine residues are shown in pink in VDW representation. PIP_2 lipids within 2.0 nm of handshake helices are shown in pink in VDW representation.

Figure S9

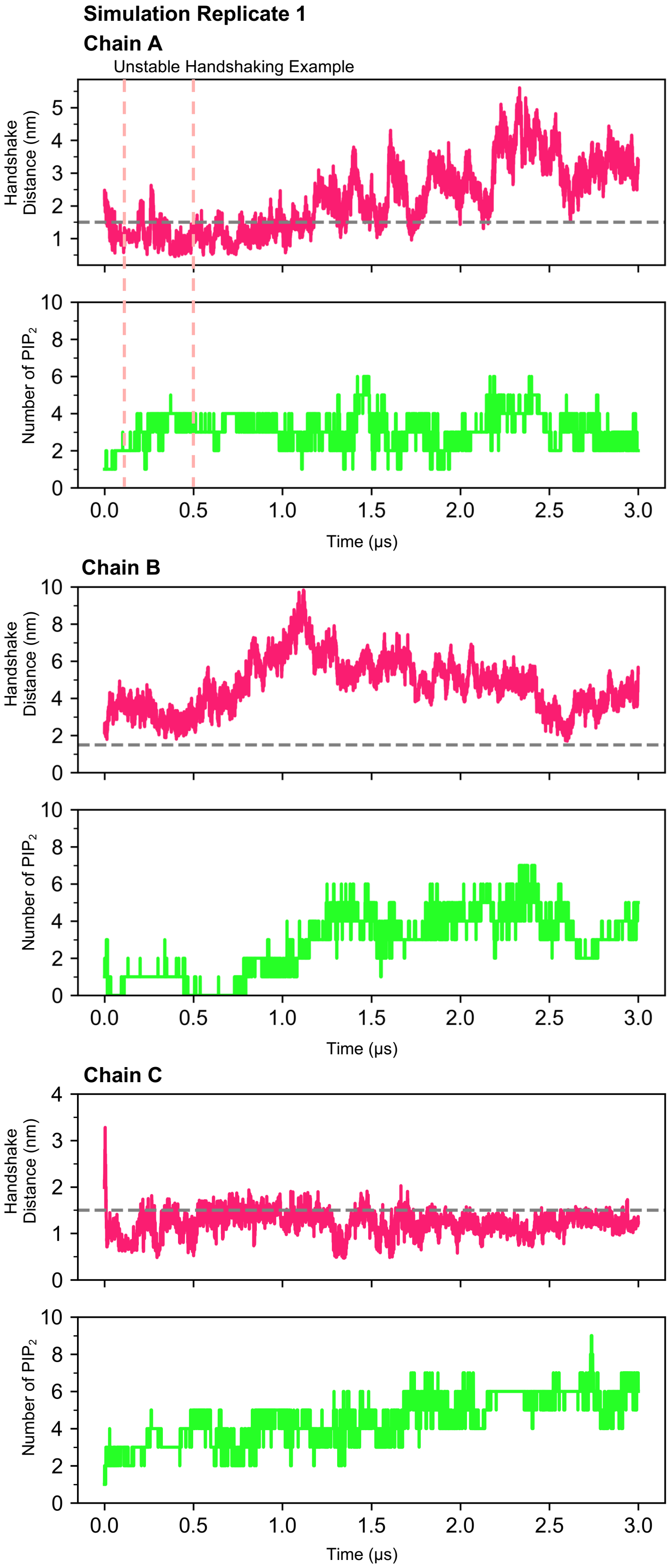

Figures S9: Additional data for Figure 3 - handshaking occurs in modes that depend on PIP_2 distribution. For simulation replicate 1, the handshake distance (between the final residues of each helix involved in the handshake interaction) and

the number of PIP_2 lipids within 2.5 nm of the SH of the interaction were plotted over simulation time for each chain of PIEZO1 (Chain labels correspond to the chain of the SH). The red dotted lines indicate the region of the plot that is shown in Figure 3.

Figure S10

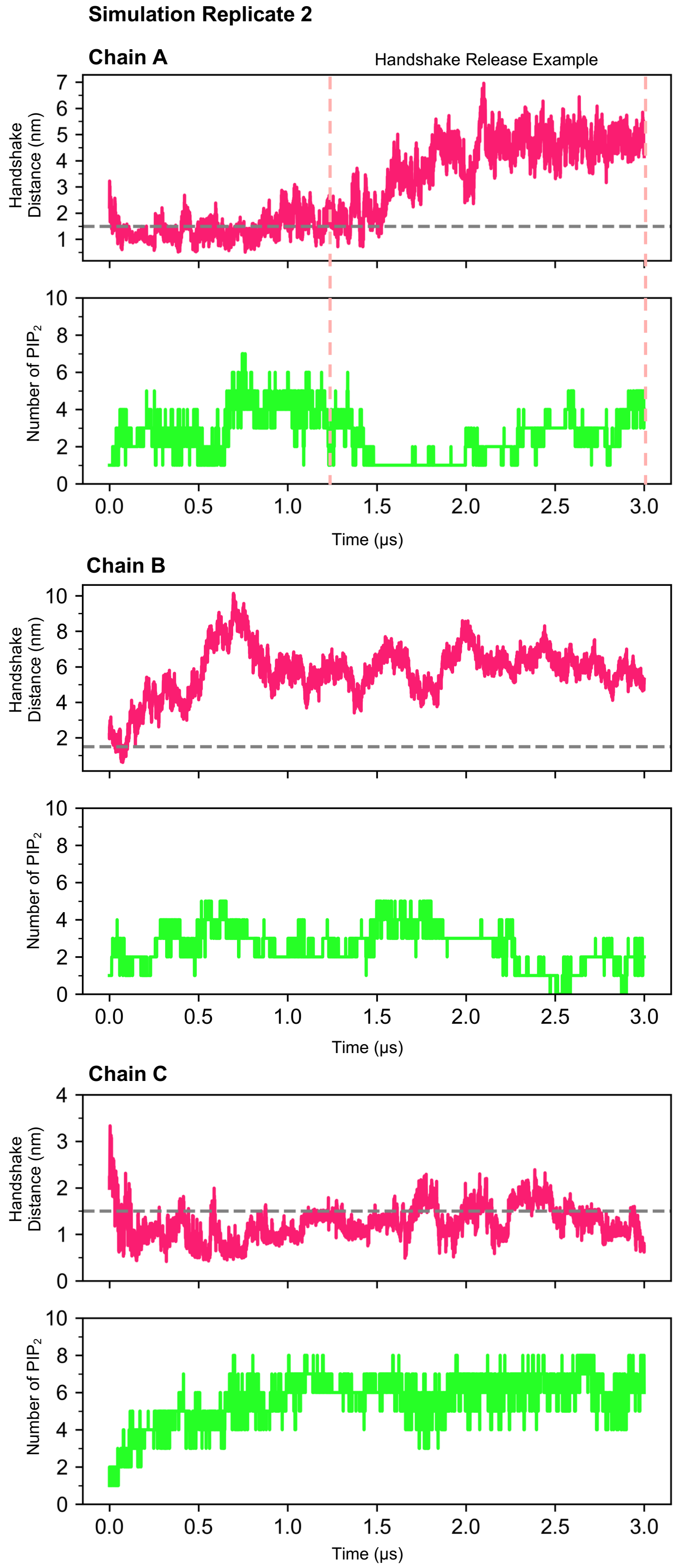

Figures S10: Additional data for Figure 3 - handshaking occurs in modes that depend on PIP_2 distribution. For simulation replicate 2, the handshake distance (between the final residues of each helix involved in the handshake interaction) and

the number of PIP_2 lipids within 2.5 nm of the SH of the interaction were plotted over simulation time for each chain of PIEZO1 (Chain labels correspond to the chain of the SH). The red dotted lines indicate the region of the plot that is shown in Figure 3.

Figure S11

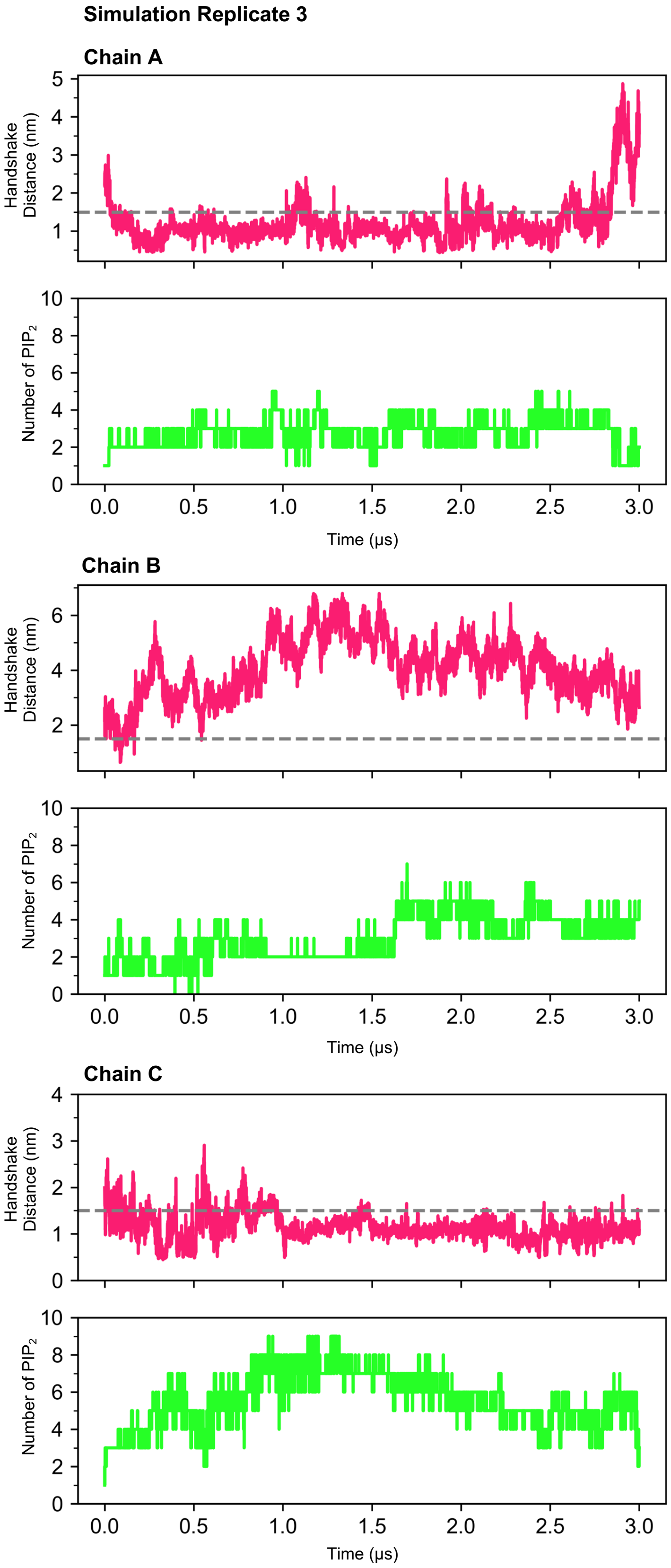

Figures S11: Additional data for Figure 3 - handshaking occurs in modes that depend on PIP_2 distribution. For simulation replicate 3, the handshake distance

(between the final residues of each helix involved in the handshake interaction) and the number of PIP_2 lipids within 2.5 nm of the SH of the interaction were plotted over simulation time for each chain of PIEZO1 (Chain labels correspond to the chain of the SH).

Figure S12

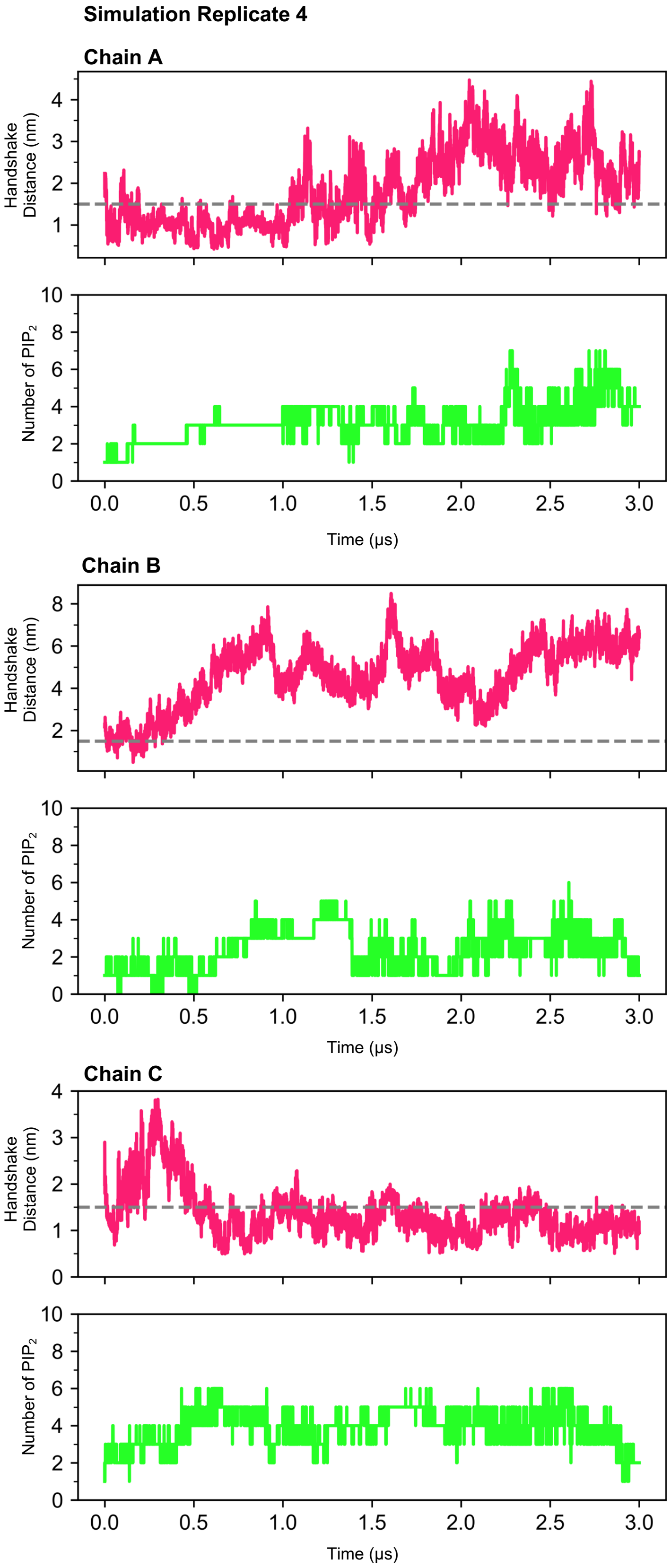

Figures S12: Additional data for Figure 3 - handshaking occurs in modes that depend on PIP_2 distribution. For simulation replicate 4, the handshake distance (between the final residues of each helix involved in the handshake interaction) and

the number of PIP_2 lipids within 2.5 nm of the SH of the interaction were plotted over simulation time for each chain of PIEZO1 (Chain labels correspond to the chain of the SH).

Figure S13

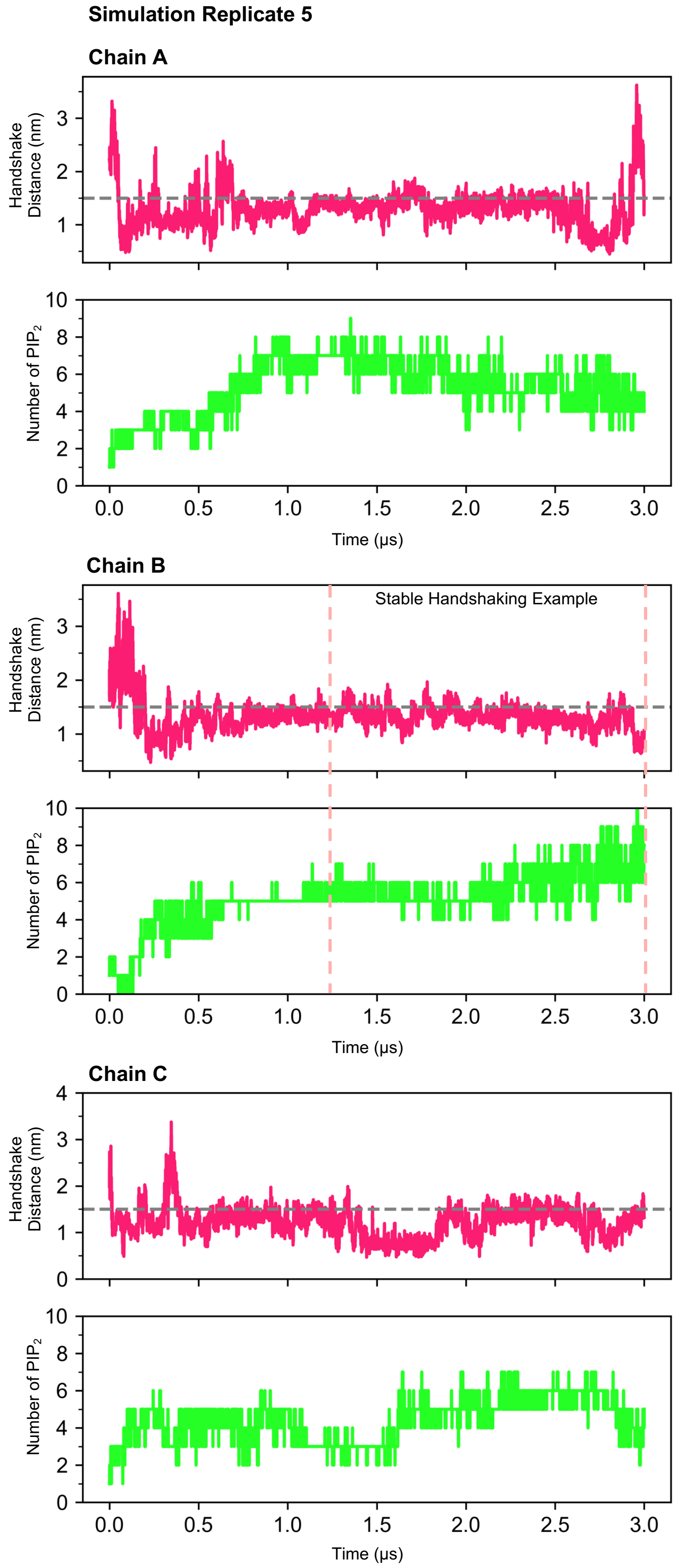

Figures S13: Additional data for Figure 3 - handshaking occurs in modes that depend on PIP_2 distribution. For simulation replicate 5, the handshake distance (between the final residues of each helix involved in the handshake interaction) and

the number of PIP_2 lipids within 2.5 nm of the SH of the interaction were plotted over simulation time for each chain of PIEZO1 (Chain labels correspond to the chain of the SH). The red dotted lines indicate the region of the plot that is shown in Figure 3.

Figure S14

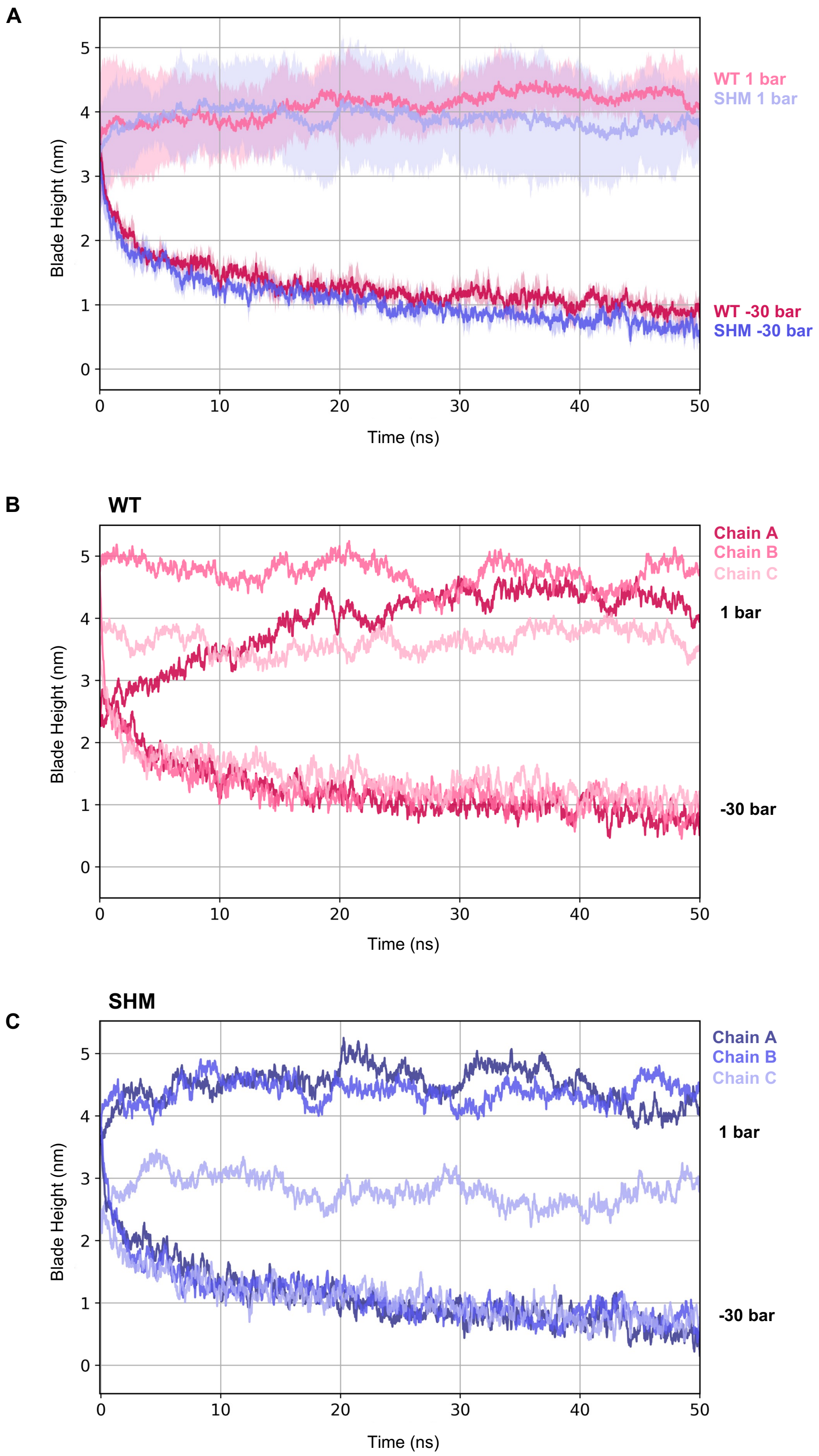

Figure S14: Analysis of blade height calculated for WT and SHM hPIEZO1 under tension. The height of hPIEZO1 blades was calculated over simulation time for WT (pink) and SHM (purple) hPIEZO1 following AT simulation in model endothelial membranes containing 5% PIP_2 in the presence of (-30 bar) and absence of (+1 bar, atmospheric pressure) tension. (**A**) Average blade heights for each system under each condition over simulation time. Blade heights were averaged between the 3 chains.

Standard deviations are plotted as transparent error bands. Data for 1 bar and -30 bar simulations are shown in light and dark colours, respectively. (**B-C**) Blade heights calculated for each chain over simulation time in the presence and absence of tension for WT hPIEZO1 (**B**) and SHM hPIEZO1 (**C**).

A Figure S15

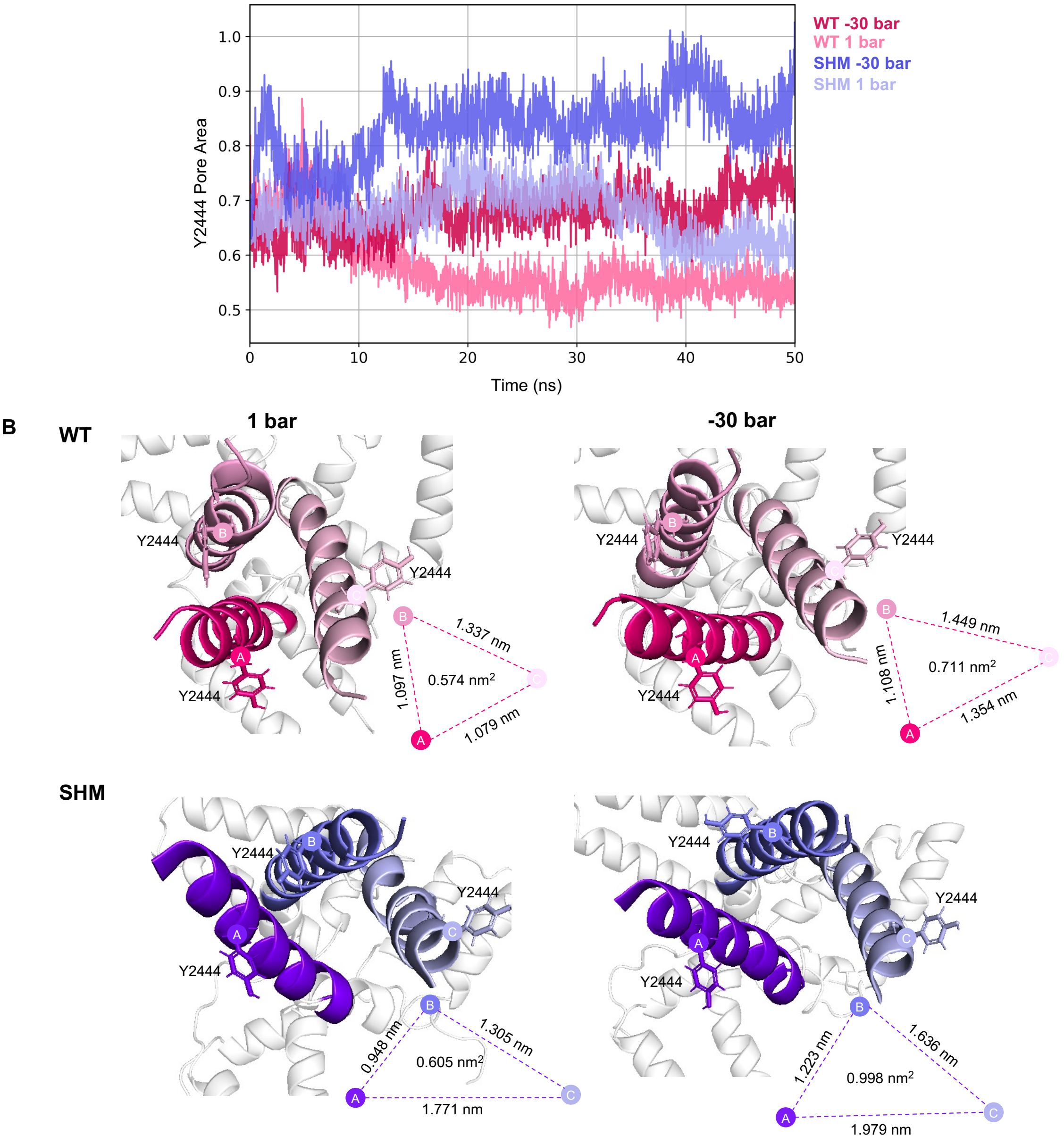

Figure S15: Analysis of changes in the pore inner helix at Y2444 under tension for WT and SHM hPIEZO1. (**A**) The area of the triangle defined by Y2444 in each chain was calculated over simulation time for WT (pink) and SHM (purple) hPIEZO1 following AT simulation in endothelial model membranes containing 5% PIP_2 in the presence of (-30 bar) and absence of (+1 bar) tension. Data for 1 bar and -30 bar tension simulations are shown in light and dark colours, respectively. (**B**) Final snapshots showing WT and SHM hPIEZO1 pore following AT simulation for 50 ns under no tension (left) and under -30 bar tension (right). Pore inner helices are shown in cartoon representation for WT (pink) and SHM (purple) hPIEZO1. Intracellular regions below the pore are transparent and shown in cartoon representation in white. Y2444 in each chain is shown in stick representation. A depiction of the triangle used to calculate the pore area at the level of Y2444 is shown in the right corner of each snapshot, with distance and area values corresponding to the final snapshot values (t= 50 ns).

Figure S16

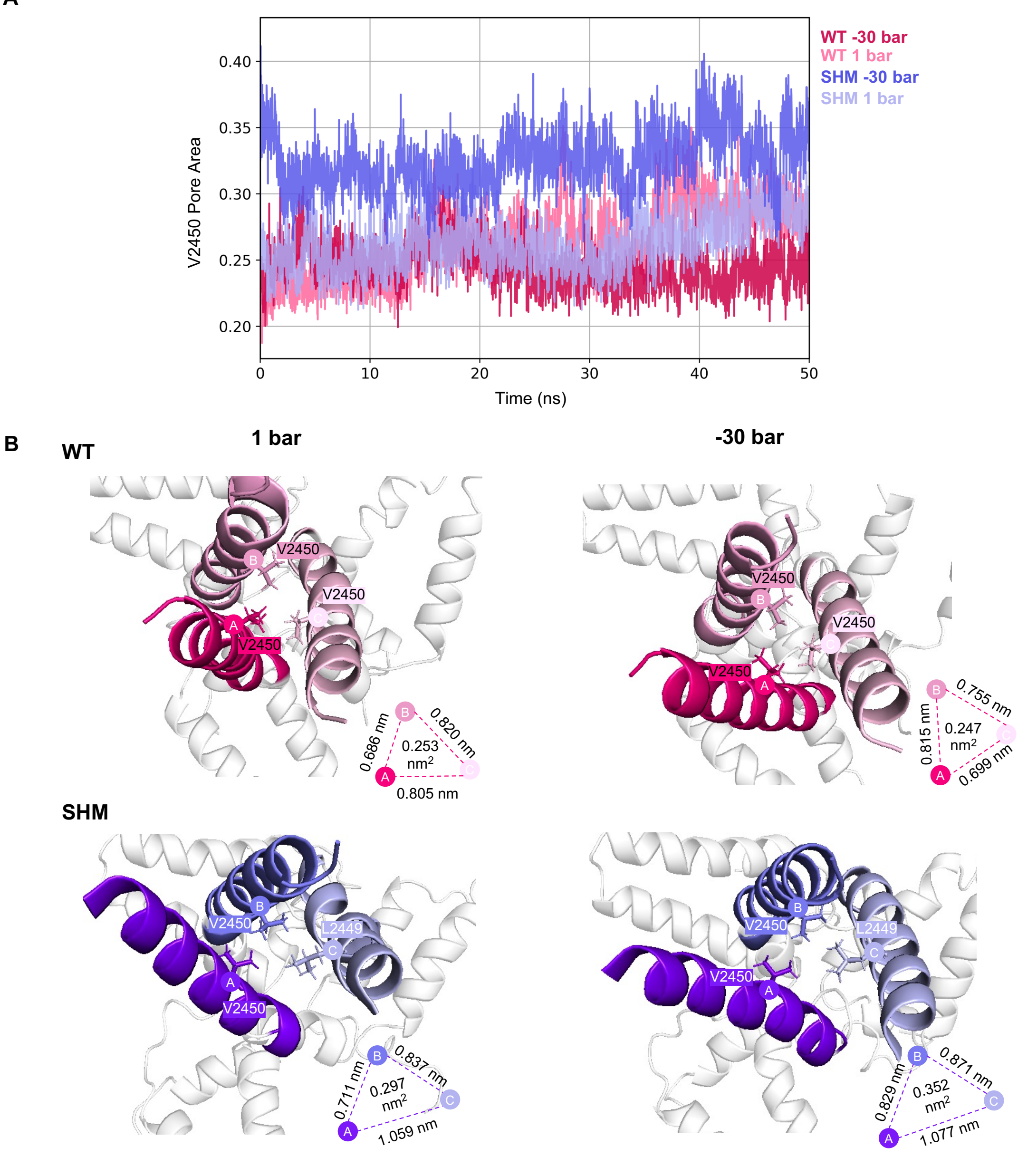

Figure S16: Analysis of changes in the pore inner helix at V2450 under tension for WT and SHM hPIEZO1. (**A**) The area of the triangle defined by V2450 in each chain for WT hPIEZO1 (red) and V2450-V2450-L2449 in SHM hPIEZO1 (blue) was calculated over simulation time following AT simulation in endothelial model membranes containing 5% PIP_2 in the presence of (-30 bar) and absence of (+1 bar) tension. Data for 1 bar and -30 bar tension simulations are shown in light and dark colours, respectively. (**B**) Final snapshots showing WT and SHM hPIEZO1 pore following AT simulation for 50 ns under no tension (left) and under -30 bar tension (right). Pore inner helices are shown in cartoon representation for WT (pink) and SHM (purple) hPIEZO1. Intracellular regions below the pore are transparent and shown in cartoon representation in white. V2450 (or L2449 in SHM hPIEZO1 chain C) in each chain is shown in stick representation. A depiction of the triangle used to calculate the pore area at the level of V2450 is shown in the right corner of each snapshot, with distance and area values corresponding to the final snapshot values (t= 50 ns).

Figure S17

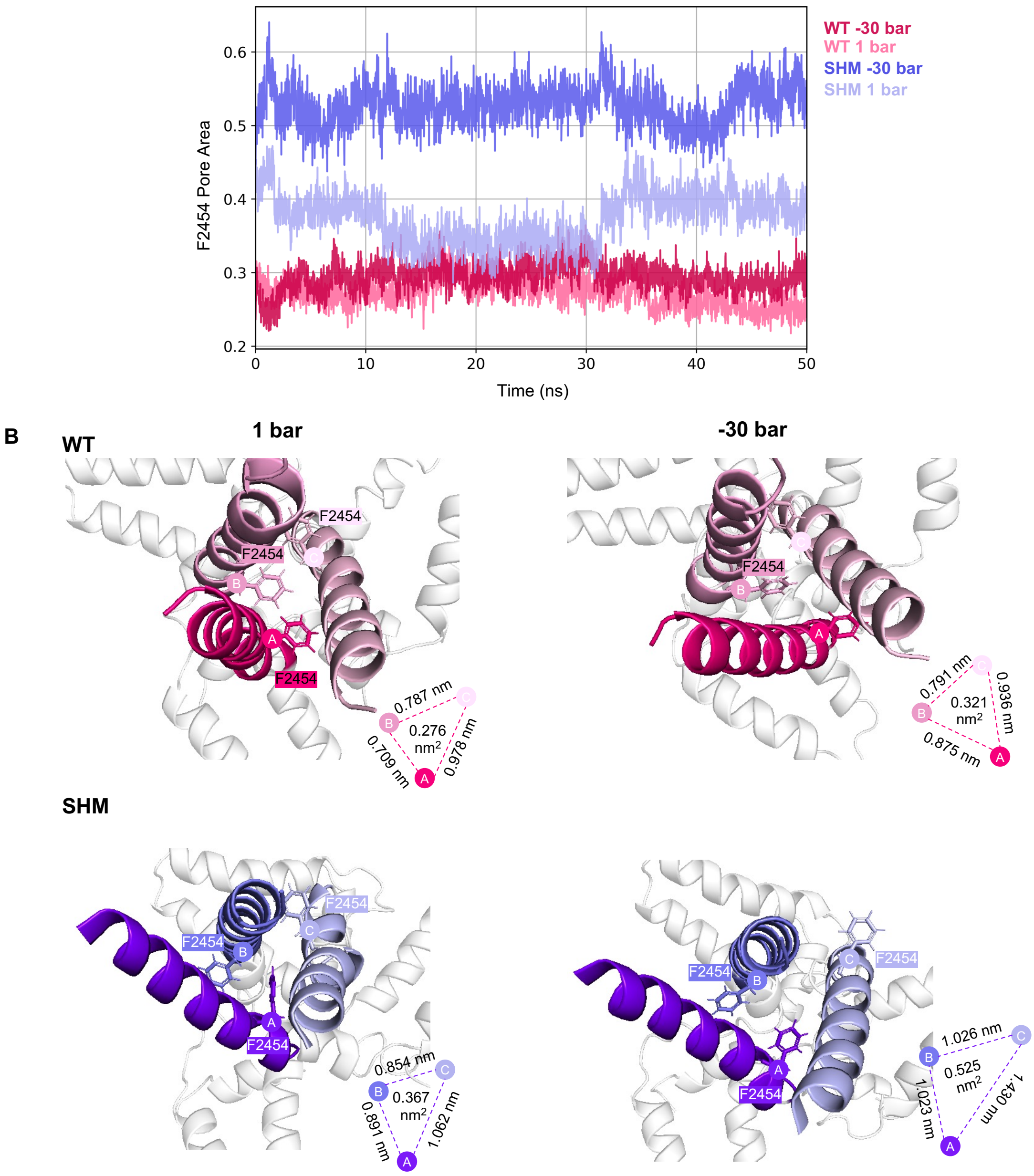

Figure S17: Analysis of changes in the pore inner helix at F2454 under tension for WT and SHM hPIEZO1. (**A**) The area of the triangle defined by F2454 in each chain was calculated over simulation time for WT (pink) and SHM (purple) hPIEZO1 following AT simulation in endothelial model membranes containing 5% PIP_2 in the presence of (-30 bar) and absence of (+1 bar) tension. Data for 1 bar and -30 bar tension simulations are shown in light and dark colours, respectively (**B**) Final snapshots showing WT and SHM hPIEZO1 pore following AT simulation for 50 ns under no tension (left) and under -30 bar tension (right). Pore inner helices are shown in cartoon representation for WT (pink) and SHM (purple) hPIEZO1. Intracellular regions below the pore are transparent and shown in cartoon representation in white. F2454 in each chain is shown in stick representation. A depiction of the triangle used to calculate the pore area at the level of F2454 is shown in the right corner of each snapshot, with distance and area values corresponding to the final snapshot values (t= 50 ns)

Figure S18

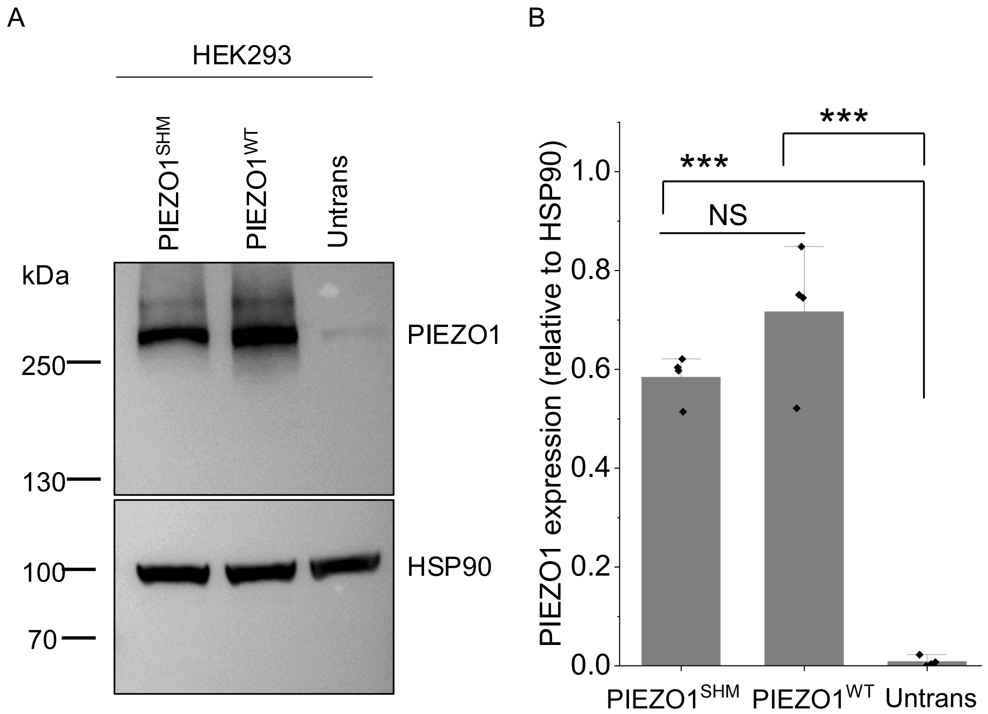

Figure S18: Western blot analysis of hPIEZO1 protein expression in HEK293 stable cell line. (**A**) Western blots showing hPIEZO1 (top) and HSP90 control protein (bottom). (**B**) Bar graph showing quantification of hPIEZO1 protein expression levels for cells expressing $hPIEZO1^{SHM}$, $hPIEZO1^{WT}$ or untransfected HEK293 cells normalised to HSP90 (n = 4). Superimposed data points are quantification of independent western blots. For the comparison between $hPIEZO1^{SHM}$ and $hPIEZO1^{WT}$ P = 0.12; $hPIEZO1^{SHM}$ and untransfected cells ***$P = 3.67\times10^{-7}$; $PIEZO1^{WT}$ and untransfected cells ***$P = 5.16\times10^{-5}$.

Figure S19

A

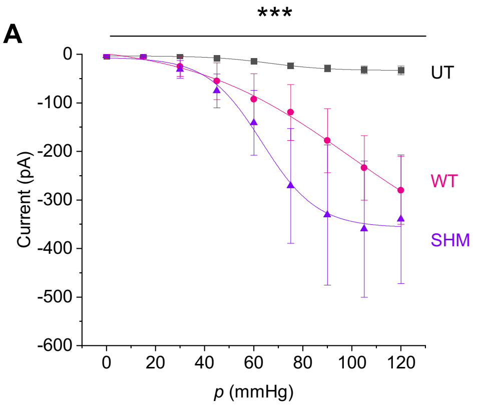

B

	p_{50} (mmHg)	d*p*
WT	98.10 ± 11.80	32.74 ± 6.70
SHM	63.36 ± 1.70	11.16 ± 1.57

C

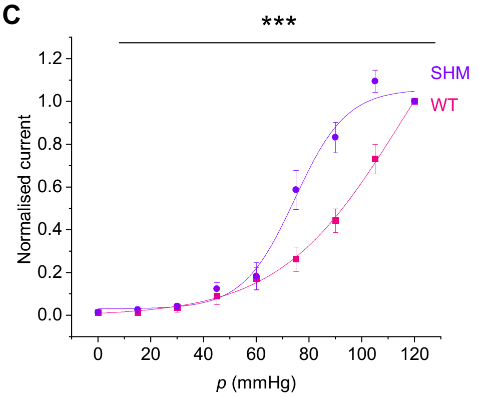

D

	p_{50} (mmHg)	d*p*
WT	115.12 ± 7.93	23.25 ± 2.65
SHM	74.58 ± 2.28	9.84 ± 2.05

E F

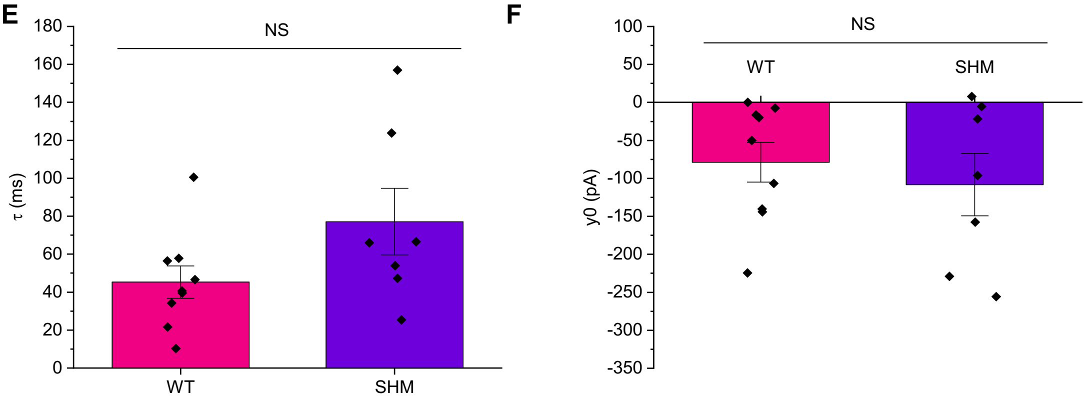

Figure S19: Additional analysis of Figure 5 patch-clamp data. (**A**) Data of Figure 5C displayed as mean ± s.e.mean absolute peak current of hPIEZO1 (WT n = 15, SHM n = 13, UT n = 19) with fitted Boltzmann functions. F-test statistical comparison of WT and SHM yielded ***P < 0.001 ($P = 1.97 \times 10^{-7}$). (**B**) Boltzmann parameters from (**A**). (**C**) WT and SHM data of (A) displayed as mean ± s.e.mean current normalised to maximum current obtained at 120 mmHg and with fitted Boltzmann functions. F-test statistical comparison of WT and SHM yielded ***P < 0.001 ($P = 2.61 \times 10^{-5}$). (**D**) Boltzmann parameters from (**C**). (**E, F**) Data from single exponential functions fitting to currents that inactivated during the 90 mmHg pulse (WT n = 9, SHM n = 7), showing time constant (τ) values (**E**) and residual non-inactivating current amplitude (y0) (**F**). Data are mean ± s.e.mean with individual data points superimposed. Two outliers were identified by Grubbs test in the WT group and

removed. No significant (NS) differences were detected by unpaired t-test not assuming equal variance (**E** P = 0.13768, **F** P = 0.55813).

Figure S20

A

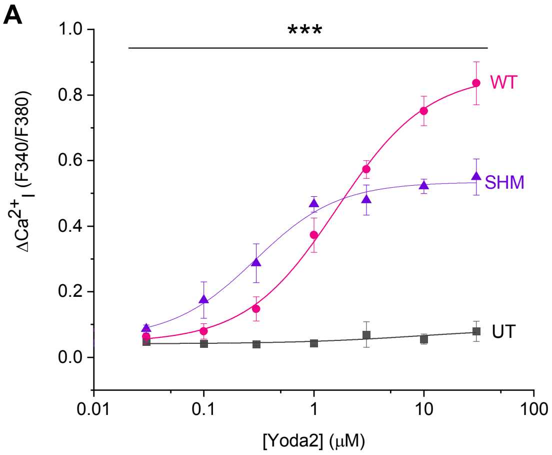

B

	EC_{50} (μM)	Hill coefficient
WT	1.62 ± 0.18	1.05 ± 0.12
SHM	0.29 ± 0.06	1.15 ± 0.26

Figure S20: Additional analysis of Figure 5 Ca^{2+} data. (**A**) Data of Figure 5E displayed as mean ± standard deviation for all 5 independent experiments with fitted Hill functions for the hPIEZO1. F-test statistical comparison of WT and SHM yielded ***$P < 0.001$ ($P = 7.285 \times 10^{-6}$). (**B**) WT and SHM Hill parameters from (**A**).

Figure S21

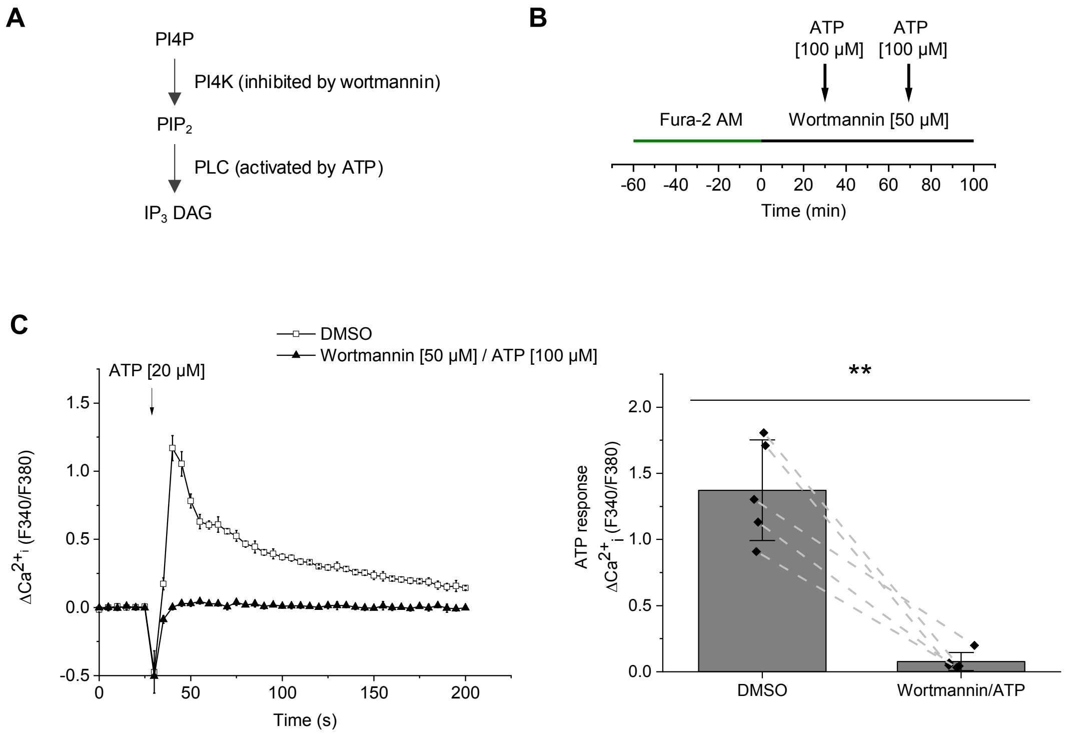

Figure S21: PIP$_2$ depletion strategy and its validation. (**A**) Schematic representation of the PIP$_2$ depletion strategy. PI4P, phosphatidylinositol 4-phosphate. PI4K, phosphatidylinositol 4-kinase. PIP$_2$, phosphatidylinositol 4,5-bisphosphate. ATP, adenosine triphosphate. PLC, phospholipase C, IP$_3$, inositol 1,4,5-trisphosphate. DAG, diacylglycerol. PI4K catalyses the conversion of PI4P to PIP$_2$. PI4K is inhibited by 50 µM wortmannin, thus it inhibits the formation of new PIP$_2$. ATP stimulates PLC activity via G protein coupled receptors, thus it depletes PIP$_2$ and generates IP$_3$, which triggers the release of Ca^{2+} from intracellular stores. (**B**) Protocol for incubating cells with the Ca^{2+} indicator dye precursor (Fura-2 AM) and then wortmannin with 2 pulses of ATP. (**C**) 96-well intracellular Ca^{2+} measurement data for HEK 293 cells. Left: example data, showing change (Δ) in intracellular Ca^{2+} in response to 20 µM ATP indicated by fura-2 fluorescence (F) ratio F340/F380. Data are mean ± s.e.mean for 3 wells (technical replicates) in a 96-well plate (1 independent experiment). Right: peak ΔCa^{2+} amplitudes for all experiments of the type shown on the left. Data are mean ± standard deviation with individual data points superimposed (n = 5 independent experiments each). Dashed lines join the individual data points for each paired comparison. ATP was applied to test for IP$_3$-induced Ca^{2+} release, which is expected to be prevented by PIP$_2$ depletion. Statistical test (paired t-test) probability (P) result was $^{**}P = 0.002$.

Figure S22

A

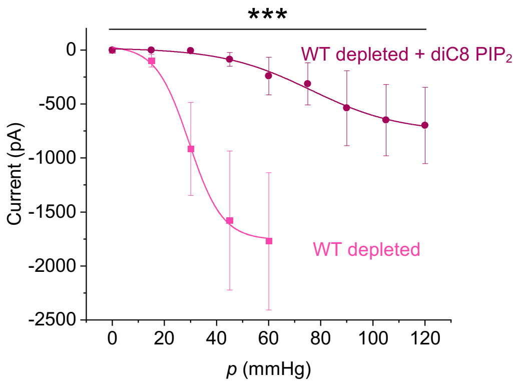

B

	p_{50} (mmHg)	dp
WT depleted	29.50 ± 1.11	6.37 ± 1.32
WT depleted + diC8 PIP_2	76.20 ± 3.52	17.10 ± 3.31

Figure S22: Additional analysis of data shown in Figure 6C. (**A**) Mean ± s.e.mean peak currents with Boltzmann functions fitted to the mean values. F-test statistical comparison of WT depleted and WT depleted + diC8 PIP_2 data yielded ***$P = 8.71 \times 10^{-8}$. (**B**) Boltzmann parameters from (**A**).

Figure S23

Handshake analysis for mPIEZO1 CG-MD simulations

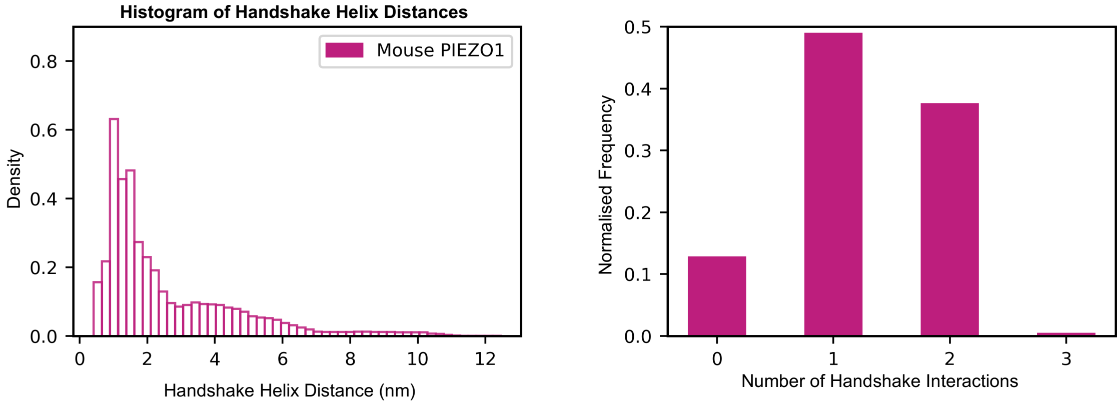

Figure S23: Analysis of interchain handshaking by mPIEZO1. Handshake analysis for mouse PIEZO1 (mPIEZO1) channel simulated in a model endothelial membrane containing 5% PIP_2, based on Chong et al (*12*). (Left) Histogram of distances between the final residue of each helix which forms the handshake interaction. (Right) Frequency of 0, 1, 2 and 3 handshake conformations.

Figure S24

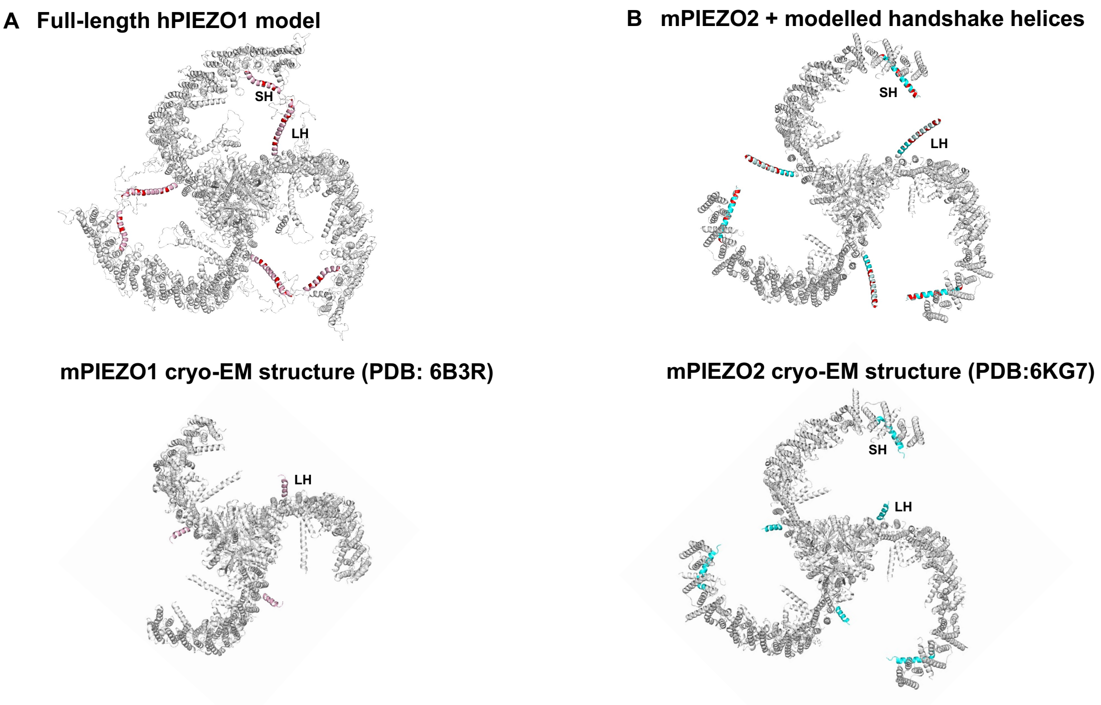

C Number of Arg and Lys residues in handshake helices

PIEZO Orthologue	Short Helix	Long Helix
Human PIEZO1	6 Arg, 0 Lys	9 Arg, 0 Lys
Mouse PIEZO1	5 Arg, 1 Lys	5 Arg, 3 Lys
Mouse PIEZO2	2 Arg, 4 Lys	2 Arg, 6 Lys

D Sequence alignment of 15 PIEZO orthologues

Short Handshake Helix

```
                                            10        20
HUMAN_PIEZO1/176-204                  LAPTRRSRLAARFRVTAHWLLVAAGRVLA
MOUSE_PIEZO1/182-210                  LATKRRLWLASRFRVTAHWLLMTSGRTLV
MOUSE_PIEZO2/199-227                  KILRRFASVASKLKEFIGNMITTAGKVVV
ARABIDOPSIS_PIEZO/141-161             HFSGIFEHLGSHLRVASCLLL--------
ATLANTIC_COD_PIEZO1/183-211           STATRAKQLAQRLRTTAHEVLQDVGRILT
BOVINE_PIEZO1/181-209                 LSPTRRSRLAARFRITAHWLLVAAGRTLA
CAT_PIEZO1/199-227                    KMFRRLASVASKLKEFIGNMITTAGKVVV
CHICKEN_PIEZO1/171-198                -TAGGDAPLSARLRVTAHWVLWAAGKGLA
DOG_PIEZO1/177-205                    QAPTRRSRLATRFQITAHWLLVAAGRTLA
KOALA_PIEZO1/181-209                  EASNRASRLAVKLKVTVHWLLKAMGNTLA
PIG_PIEZO1/176-204                    LPLKGRSRLAARFRITAHWLLVAAGRALA
RHESUS_MACAQUE_PIEZO1/176-204         LAPTRRSRLAARFRVTAHWLLVAAGRVLA
TUFTED_DUCK_PIEZO1/172-199            -AADGDTPLSARLRVTAHWLLWAAGKGLA
WESTER_EUROPEAN_HEDGEHOG_PIEZO1/1-    --MKRKSRLAARFRITAHWLLVAAGRTLA
ZEBRAFISH_PIEZO1/190-218              PSSSSAAQLAARLRATAQRFLRNMGRILA
```

Long Handshake Helix

```
                                                    10        20        30        40
HUMAN_PIEZO1/1912-1951                       RPSRSGGRVRAAGRRLQGFCLSLAQGTYRPLRRFFHDIL--H
MOUSE_PIEZO1/1927-1966                       EKSKFRERMKAAGRRLQSFCVSLAQSFYQPLQRFFHDIL--H
MOUSE_PIEZO2/2211-2250                       KRELYMEKLQEHLIKAKAFTIKKTLQIYVPIRQFFYDLI--H
ARABIDOPSIS_PIEZO/1870-1904                  KSLTPASDVAKEIRKAQHSGLGEG-------TGFPYPILSVI
ATLANTIC_COD_PIEZO1/1755-1794                QVTQDGGDRNIISCYLLPFFSSSVQDVYRPTRCFFVNIL--H
BOVINE_PIEZO1/1903-1942                      RRSRPQERVRVLGVRLQSFCLSLARSMYWPVRRFFQDIL--H
CAT_PIEZO1/2154-2193                         KRELYMEKLQEHLIKAKAFTIKKTLQIYVPIRQFFYNLI--H
CHICKEN_PIEZO1/1854-1893                     KQSHSQKKLKAFGLRVKLFFLTMAQNMYQPVRGFFHDIL--H
DOG_PIEZO1/1904-1943                         KWSRPRERMTAMGLRLQTFCLAVAQSMYRPLRRFFDDIL--H
KOALA_PIEZO1/1925-1964                       RMSRPQEKMKATGLKIRRFFKTMVQNIYQPIRRFFWDIL--H
PIG_PIEZO1/1885-1924                         RRRRPRERVRALGVWLQSVCLSLAQGAYRPLRRFFQDIL--H
RHESUS_MACAQUE_PIEZO1/1916-1955              RPSRAGRRVRVAGRQLQGFCLSLAQGTYRPLRRFFHDIL--H
TUFTED_DUCK_PIEZO1/1889-1928                 KQSHSQKKLKALGLRVKLFFLTVAQNTYQPVRGFFRDIL--H
WESTERN_EUROPEAN_HEDGEHOG_PIEZO1/1699-17     QRIRPKDRVRAAGLWLQGFCLSLAQGIYQPLQHFFHDIL--H
ZEBRAFISH_PIEZO1/1929-1966                   --ETSKKLLNAIEGKFKSLFLSVVKNVYRPTWDFFQNIL--H
```

Figure S24: Structures of different PIEZOs and sequence conservation in putative handshake helices. (**A**) Full-length hPIEZO1 structural model shown from top view in cartoon representation (top). Handshake helices are coloured in pink, with

the positively charged arginine residues coloured red. The rest of the channel is coloured grey. mPIEZO1 cryo-EM structure (PDB: 6B3R)(*9*) shown from top view in cartoon representation (bottom). Regions of handshake helices resolved by cryo-EM coloured in pink. The rest of the channel is coloured grey. (**B**) Mouse PIEZO2 structure with modelled handshake helices shown from top view in cartoon representation (top). Handshake helices were partially resolved by cryo-EM (shown in cyan), the cryo-EM structure (PDB:6KG7)(*20*) is shown from top view in cartoon representation (bottom). The full helices were modelled based on secondary structure prediction and the modelled regions shown in pale cyan. Positively charged arginine and lysine residues, on the full handshake helices, are coloured red and the rest of the channel is shown in grey. (**C**) Numbers of arginine and lysine residues in the short and long handshake helices for hPIEZO1, mPIEZO1 and mPIEZO2. (**D**) Sequence alignment of 15 PIEZO orthologues. Alignment is shown for the short handshake helix residues (residues 176-203 in hPIEZO1) (top) and long handshake helix residues (residues 1912-1951 in hPIEZO1) (bottom). Arginine and lysine residues have been highlighted in red and pink respectively. 15 PIEZO orthologues were selected: Human PIEZO1 (Uniprot: Q92508), Mouse PIEZO1 (Uniprot: E9PUQ9), Mouse PIEZO2 (Uniprot: Q8CD54), Dog (Uniprot: A0A8I3MUY4), Zebrafish (Uniprot: A0A8N7TDV6), Pig (Uniprot: A0A480F0E9), Tufted Duck (Uniprot: A0A6J3DPG2), Bovine (Uniprot: F1MD64), Cat (Uniprot: A0A337SVB0), Koala (Uniprot: A0A6P5KK49), Western European Hedgehog (Uniprot: A0A1S3WLZ8), Rhesus Macaque (Uniprot: A0A5F7ZT10), Chicken (Uniprot: A0A8V0Y724), Atlantic Cod (Uniprot: A0A8C4YZ71), Arabidopsis thaliana (Uniprot: F4IN58).

Figure S25

Atomistic Simulations (+1 bar)

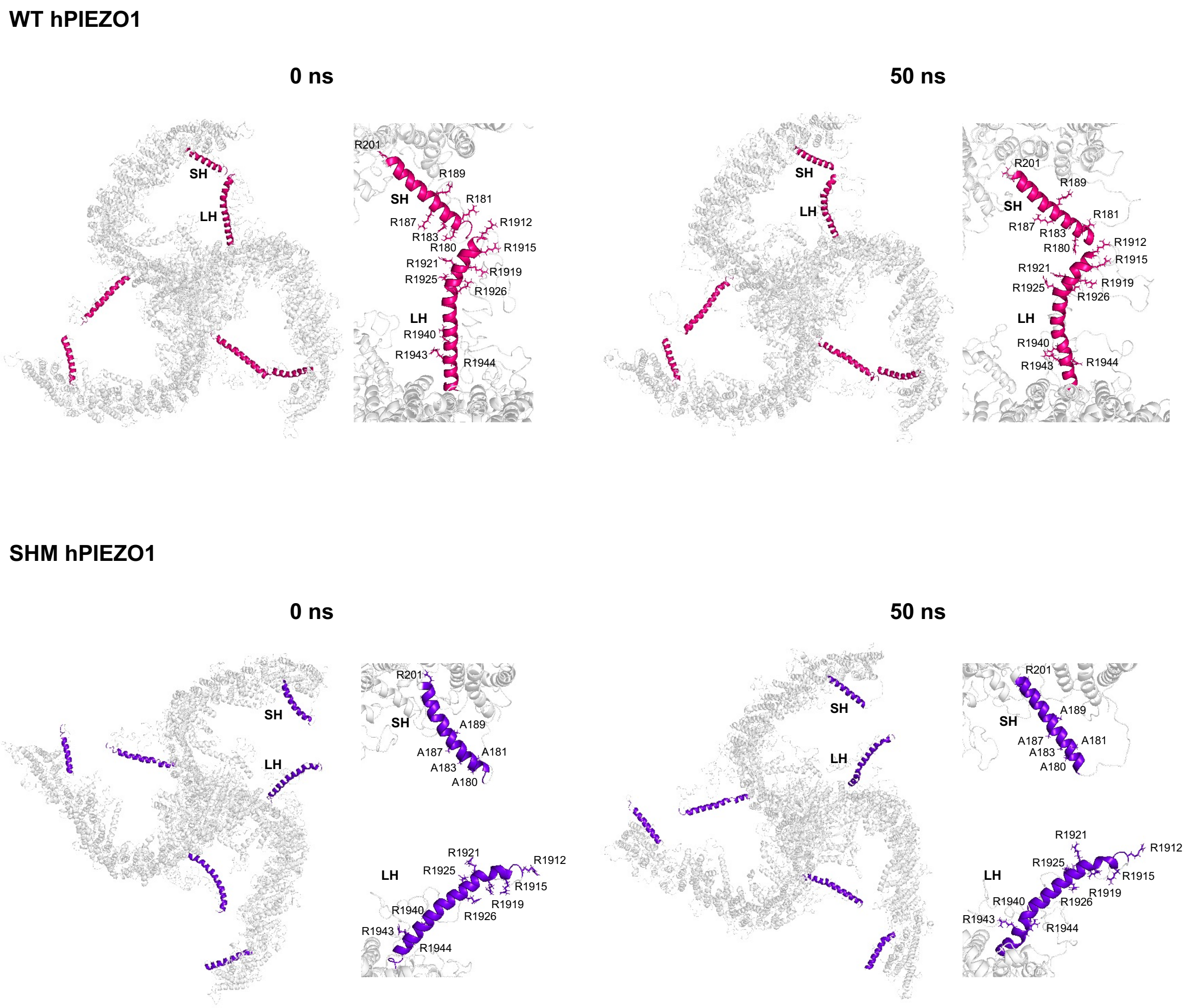

Figure S25: Analysis of hPIEZO1 handshake structures following simulation at atomistic (AT) resolution at +1 bar. WT (top) and SHM (bottom) hPIEZO1 structures extracted from simulation trajectories at time = 0 ns and 50 ns following AT simulation under no tension. Structures are shown from the top view (left) in cartoon representation. The handshake helices are coloured pink (WT) and purple (SHM). The rest of the channel is coloured grey. Handshake helices are shown in more detail (right) with arginine residues (or mutated alanine in SHM) side chains shown in stick representation. Handshake helices retain alpha-helix structures following AT simulation.

Figure S26

A

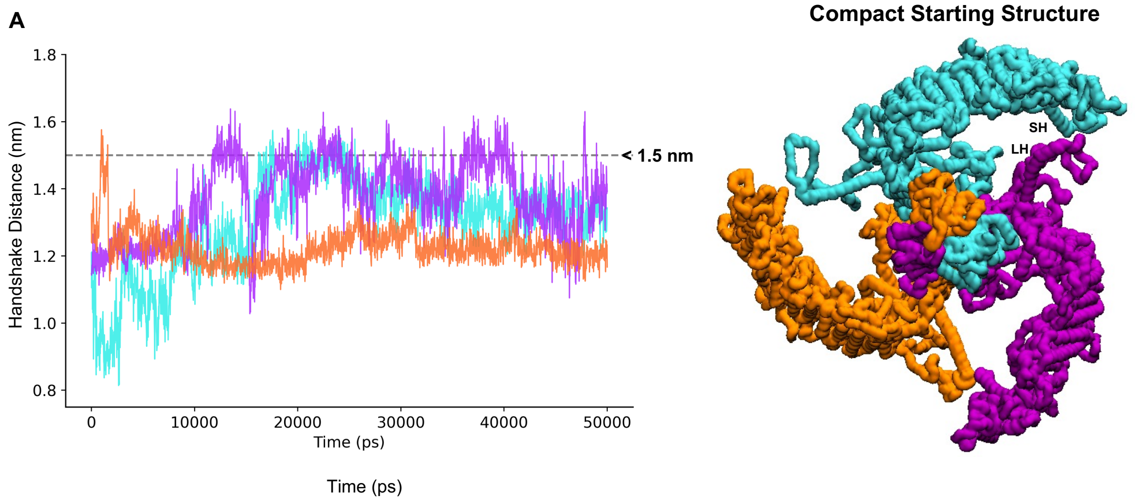

B

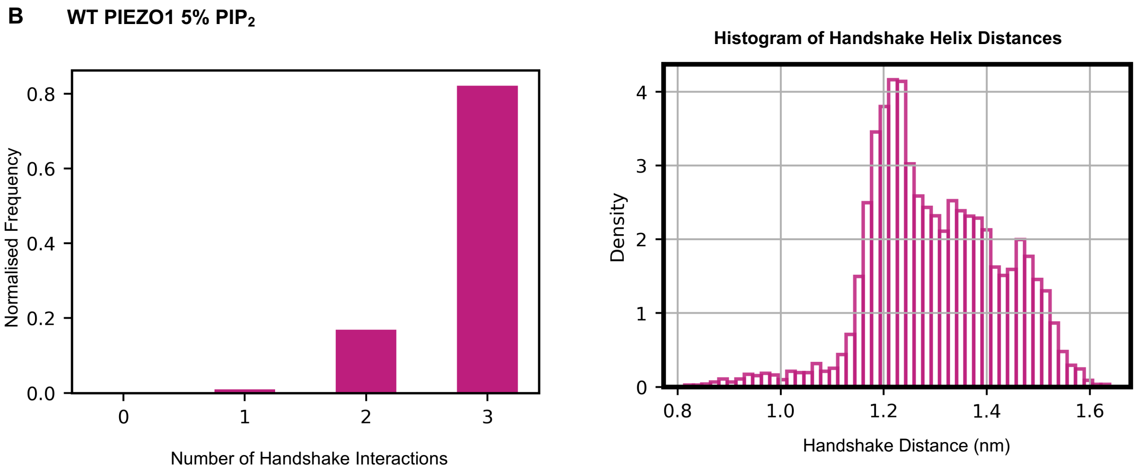

Figure S26: Analysis of interchain handshaking by WT hPIEZO1 simulated in an endothelial model membrane at atomistic (AT) resolution from a compact starting structure. (**A**) Distances calculated over simulation time between the final residue of each helix which forms the handshake interaction for each chain (left). Starting structure of WT PIEZO1 backbone shown in surface representation. Chains are shown in cyan, orange and purple. (**B**) Handshake analysis. Frequency of handshake states formed during simulation time (left). Histogram of distances between the final residue of each helix which forms the handshake interaction (right).

Figure S27

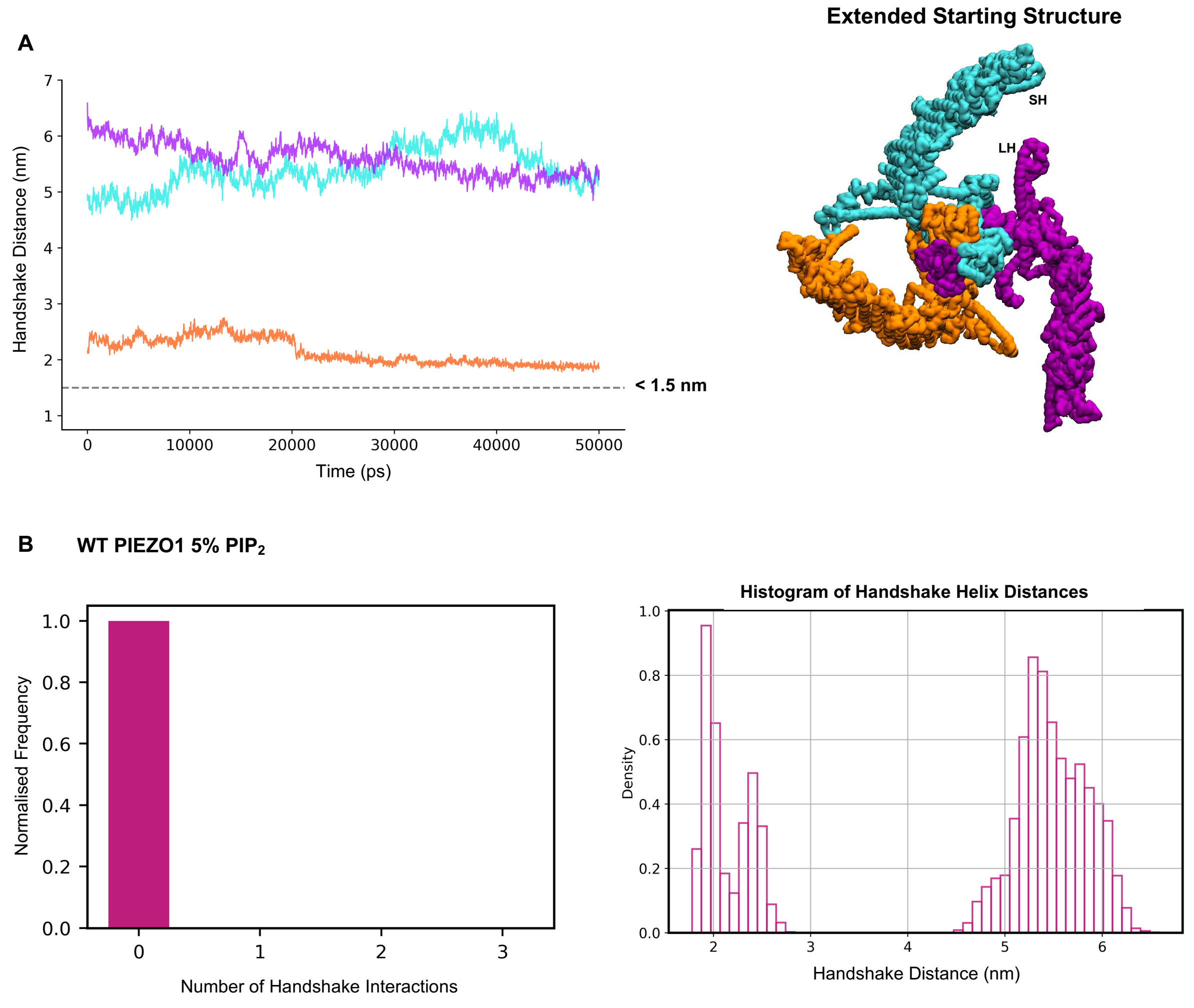

Figure S27: Analysis of interchain handshaking by WT hPIEZO1 simulated in an endothelial model membrane at atomistic (AT) resolution from an extended starting structure. (**A**) Distances calculated over simulation time between the final residue of each helix which forms the handshake interaction for each chain (left). Starting structure of WT PIEZO1 backbone shown in surface representation. Chains are shown in cyan, orange and purple. (**B**) Handshake analysis. Frequency of handshake states formed during simulation time (left). Histogram of distances between the final residue of each helix which forms the handshake interaction (right).

Figure S28 **PIP_2 Lipid Contacts**

Compact Starting Structure

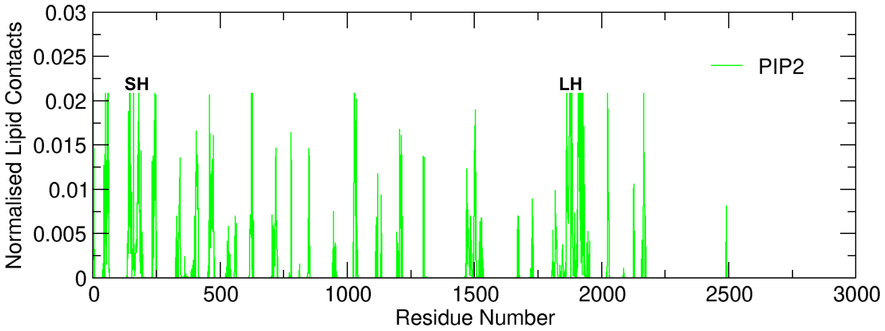

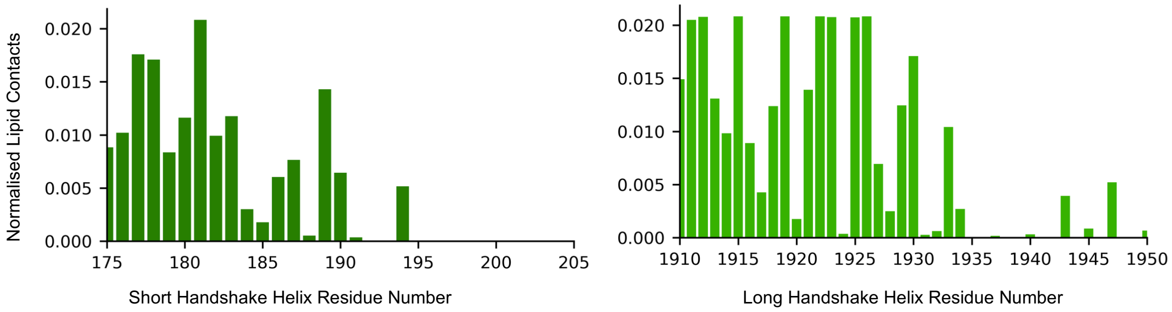

Extended Starting Structure

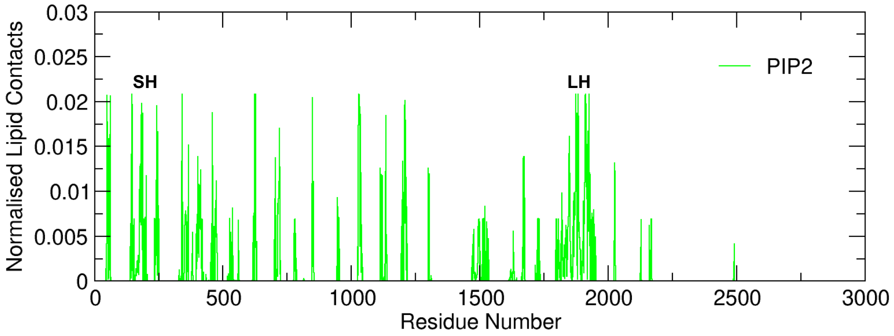

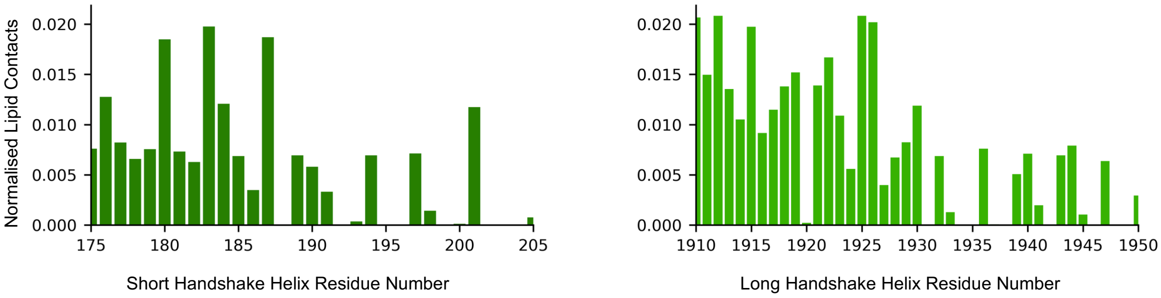

Figure S28: Lipid contacts analysis of WT hPIEZO1 residues with PIP_2 lipids following simulation at atomistic (AT) resolution from compact and extended starting structures. Lipid contacts were normalised for the number of frames and the number of PIP_2 lipids. Lipid contacts were averaged across the 3 hPIEZO1 subunits. Lipid contacts with SH and LH of handshake interaction are shown in more detail.

Video S1: PIP_2 mediates PIEZO1 handshake concept. Video showing PIEZO1 handshake interaction during simulation trajectory of hPIEZO1 simulated in an endothelial model membrane containing 5% PIP_2. hPIEZO1 model is shown in surface representation and is coloured white. When a handshake interaction forms the SH and LH change colour to red. PIP_2 lipids are shown in VDW representation in pink.
